# 1,6-Diazapyrene: A Novel, Well-Defined, Small-Size Prototype System for Nitrogen-Containing PAHs

**DOI:** 10.1021/acs.jpca.5c01474

**Published:** 2025-05-12

**Authors:** Indranil Bhattacharjee, Liangxuan Wang, Nerea Gonzalez-Sanchis, Begoña Milián-Medina, Rafael Ballesteros, Reinhold Wannemacher, Rafael Ballesteros-Garrido, Johannes Gierschner

**Affiliations:** † Madrid Institute for Advanced Studies, IMDEA Nanoscience, C/Faraday 9, Ciudad Universitaria de Cantoblanco, Madrid 28049, Spain; ‡ Institute of Physical and Theoretical Chemistry, Eberhard Karls University Tübingen, Tübingen 72076, Germany; § Department for Organic Chemistry, Faculty of Chemistry, 16781 University of Valencia, Burjassot, Valencia 46100, Spain; ∥ Department for Physical Chemistry, Faculty of Chemistry, University of Valencia, Burjassot, Valencia 46100, Spain

## Abstract

The quest for nitrogen-doped (N-doped) polycyclic aromatic hydrocarbons (PAHs) requires well-defined prototype systems to understand the relationship between the structure and the resulting photophysical and photochemical properties. To this end, a novel, simple, and small compound, 1,6-diazapyrene, was synthesized. In-depth analysis, employing optical spectroscopy and (time-dependent) density functional theory, (TD-)­DFT, elucidates the optical excitations on the basis of MO symmetry, energy, and topology considerations; the study further unveils the photophysical and photochemical deactivation kinetics after photoexcitation, revealing extreme changes against pyrene as well as against the well-known 2,7-diazapyrene isomer. The high sensitivity of the aza-substitution position to generate such changes is considered as highly relevant for the targeted design of N-doped PAHs in general.

## Graphical abstract

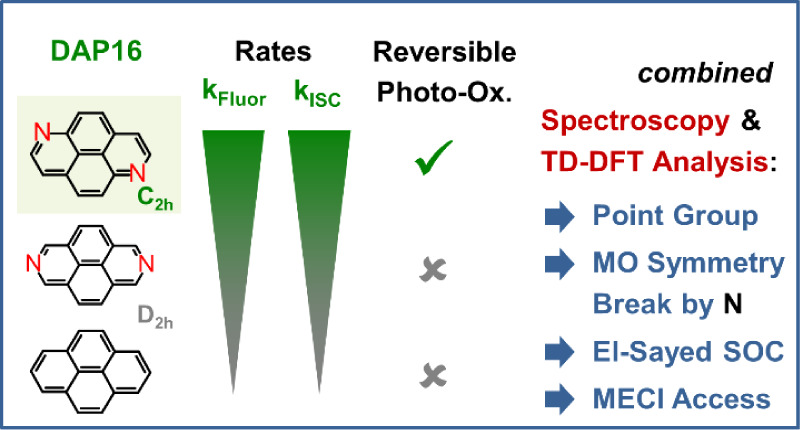

Nitrogen-doped (N-doped) polycyclic aromatic hydrocarbons (PAHs) have found much interest in the past years to tune the electronic, optical and photophysical properties of PAHs, graphene, and carbon dots in (opto)­electronic, (photo)­catalytic and biochemical and biomedical applications.^1–6^ Nevertheless, the modulation of the properties depends significantly on the type of PAH annulation (linear, angular, peri-annulated) and the number of annulated rings, as well as on the type of N-doping (pyridinic, pyrrolic, graphitic) and the number and positioning of N atoms.^1^ The complexity of possible manifestations of N-PAHs demands the investigation of prototype systems, for instance by systematic structure variation,^6–11^ or computational screening of small model systems; in particular, for the latter, pyrene (**Py**) was used as a paradigmatic platform for N-doping.^4^ This is *inter alia* connected with the outstanding position of **Py** in materials and life science applications,^12,13^ as well as serving as a prolific synthon to create functional dyes.^14–17^ In any case, little attention was paid to the detailed understanding of N-doping of **Py** for photophysical and photochemical deactivation pathways, although this is of central importance for the functionality in the various applications. This is done in the present study, on a novel, small-size prototype system, without bearing any substitutes other than hydrogen atoms, that is 1,6-diazapyrene (**DAP16**), being a long-imagined, but until now not synthesized DAP isomer.

In fact, somewhat surprisingly, out of the 15 possible neutral DAP isomers^4,18–21^ only five have been synthesized (i.e., 1,3-, 2,7-, 4,5-, 4,9-, 4,10-DAP).^18^ Among these, in particular derivatives of the 2,7-isomer (**DAP27**) were studied for the purpose of organic electronics,^1^ intercalation of nucleic acids,^20–22^ sensors,^21^ building blocks for metal–organic frameworks^21^ and molecular machines,^21,23^ as well as model systems for N-PAHs and carbon dots.^4^ In the case of solvothermally synthesized carbon dots it has actually been shown that N-PAHs are responsible for their photoluminescence, even though the presence of specific DAPs has not been detected.^24^ Although it is known from computational studies that the UV–vis spectra may significantly change upon structural variation in DAP isomers,^4^ the changes in photophysics and -chemistry upon structure variation have not been studied so far. This is, however, of crucial importance for the application of N-PAHs. In fact, in comparison with **Py** and **DAP27**, the new **DAP16** isomer exhibits enormous changes of the optical and photophysical properties, providing violet fluorescence with a (moderately high) quantum yield of Φ_F_ = 34% with sub-ns decay time (τ_F_ = 0.73 ns) in comparison with up to 400 ns in **Py** ^25,26^ and 10 ns in **DAP27**.^27^ Furthermore, upon intense UV laser irradiation, complete, reversible photooxidation of **DAP16** is observed; in all, this clearly evidences the need for a detailed understanding of the excited state properties and deactivation properties.

Such in-depth investigation is performed in the current work on the novel compound **DAP16**, combining detailed steady-state and time-resolved photoluminescence spectroscopy with (time-dependent) density functional theory, (TD) DFT, to fully rationalize the optical excitations on the basis of MO symmetry, energy and topology considerations. TD-DFT further tracks the observed photophysical and -chemical deactivation kinetics after photoexcitation, revealing the origin of the differences to **DAP27** and **Py**. Overall, **DAP16** is identified as the smallest possible structural change in the **Py** core, which leads to these dramatic alterations of the photophysics and photochemistry. The extreme sensitivity of the aza-substitution position to generate such changes is thought to be of utmost relevance for the targeted design of N-PAHs in general.

## Experimental and Computational Details

**DAP16** was synthesized by acceptorless dehydrogenative condensation^28,29^ from naphthalene-1,5-diamine (1 mmol) and ethylene glycol (5 mL) employing both Pt/Al_2_O_3_ (1.7 mol %) and ZnO (4.5 mol %) as catalyst at 175 °C. As shown in Scheme 1, this reaction provided an almost equimolecular mixture of **DAP16** and 3,8-dihydroindolo­[7,6-*g*]­indole **BI­[7,6-** *g* **]**, and fully characterized by ^1^H-, ^13^C NMR and HRMS (high resolution mass spectrometry). This glycol/aniline methodology has been employed in the past for the preparation of many different indole derivatives.^30,31^ Aromatic amines are prone to react at the *ortho* position creating five membered rings under these conditions.^32^ However, the particular structure of naphthalene-1,5-diamine allows a different, and unreported, reaction path at the *peri* position which allows the formation of the six membered ring. **DAP16** could be identified due to the *J* ^H2H3^ ∼ 5 Hz coupling constant, this value is typically observed between H2 and H3 in pyridines. In contrast, **BI­[7,6-** *g* **]** presented well reported signals from indole core including broad NH peak around 12 ppm. **BI­[7,6-** *g* **]** can be removed from the reaction mixture by trituration in chloroform due to its limited solubility. Pure samples of **DAP16** can be obtained by chromatography or by sublimation.

**Scheme 1:**
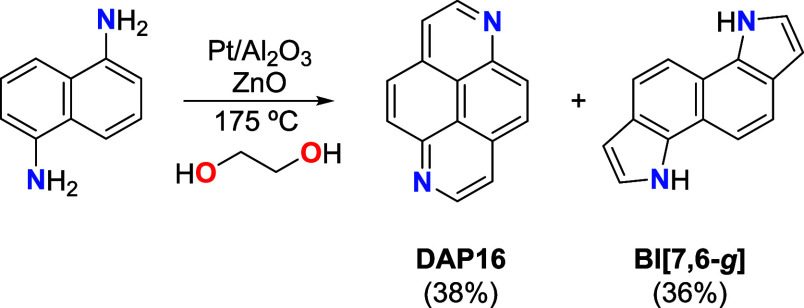
Synthesis of **DAP16**

Analysis of **DAP16**: pale yellow solid, (77 mg, 38%). Mp 223–224 °C, ^1^H NMR (300 MHz, CDCl_3_) δ 9.34 (d, *J* = 5.2 Hz, 2H), 8.51 (d, *J* = 9.2 Hz, 2H), 8.31 (d, *J* = 9.2 Hz, 2H), 8.13 (d, *J* = 5.1 Hz, 2H). ^13^C NMR (75 MHz, CDCl_3_) δ 148.7 (2xC), 147.8 (2xCH), 135.0 (2xC), 133.5 (2xCH), 129.6 (2xCH), 119.6 (2xCH), 118.9 (2xC). HRMS (ESI-TOF) *m*/*z*: [M^+^] calculated for C_14_H_8_N_2_ 205.0760; found: 205.0758. Analysis of **BI­[7,6-** *g* **]**: black solid (74 mg, 36%). Mp 207–208 °C ^1^H NMR (300 MHz, DMSO-d_6_) δ 11.80 (s, 2H), 7.96 (d, *J* = 8.5 Hz, 2H), 7.73–7.66 (m, 2H), 7.38–7.32 (m, 2H), 6.58 (dd, *J* = 3.0, 1.9 Hz, 2H). ^13^C NMR (75 MHz, DMSO-d_6_) δ 132.3­(2xC), 123.0 (2xCH), 122.9 (2xC), 119.9 (2xCH), 118.0 (2xC), 113.3 (2xCH), 103.1 (2xCH). HRMS (ESI-TOF) *m*/*z*: [M – H^+^] calculated for C_14_H_10_N_2_ 205.0760; found: 205.0759. For further details, see text and Figures S1–S12.

For the spectroscopic investigations at room temperature (RT; 22 °C), **DAP16** was dissolved in dichloromethane (DCM; *c* = 5 × 10^–6^ M) of spectroscopic quality (Uvasol, Sigma-Aldrich). Absorption spectra were recorded on an Agilent Technologies Cary 60 UV/vis absorption spectrometer. Fluorescence spectra were obtained with a Horiba FluoroMax-4 spectrofluorometer; spectra were corrected for the wavelength dependence of the detection unit. The fluorescence quantum yield of **DAP16** was determined against quinine sulfate dihydrate as a standard (Φ_F,st_ = 0.59).^33^ Fluorescence lifetimes were measured by time-correlated single photon counting (TCSPC) using a 320 nm pulsed LED (pulse width 933.6 ps) at 10 MHz repetition rate (Edinburgh Instruments) as excitation source, and a HydraHarp 400 (Picoquant) multichannel time correlator. Photons emitted at a particular wavelength were thereby detected with a thermoelectrically cooled Hamamatsu photomultiplier coupled to a *f* = 0.5 m spectrometer (Acton SP2500, Princeton Instruments) equipped with a 600 lines per mm grating blazed at 500 nm. The fluorescence decay curves were fitted via reconvolution with the instrumental response function (IRF) using the EasyTau software (PicoQuant). For phosphorescence measurements, samples of **DAP16** doped in poly­(methyl methacrylate), PMMA (0.1 wt %), were mounted in a Magnex Scientific needle valve optical cryostat operated with liquid nitrogen. The nitrogen was pumped down to 65 K and allowed to warm up slowly to remove bubbles; for further details on the setup, see Supporting Information. For photooxidation experiments, all the samples were dissolved in DCM (*c* = 5 × 10^–6^ M), irradiated with a 355 nm Nd:YAG laser (pulse energy 20 μJ, repetition rate 1 kHz, beam diameter 3 mm at the cuvette) under continuous stirring for several minutes and absorption and emission spectra were periodically recorded using an Agilent Technologies Cary 60 UV/vis absorption spectrometer and a Horiba FluoroMax-4 spectrofluorometer, respectively. For the comparison with **Py** and **DAP27**, we relied on available literature data;^26,27^ nevertheless, we remeasured the spectra of **Py** in DCM (purchased from Acros organics; purity 98%). For comparative photooxidation studies with **DAP16**, **DAP27** was resynthesized following the protocol of ref. 33. The absorption spectrum of the product, however, turned out to be of inferior quality compared to the former report,^27^ so that to compare the spectral properties, we relied on the latter; although this was measured in H_2_O, the moderately small solvent shifts observed were irrelevant for the qualitative comparison of the three compounds below.

DFT geometry optimizations were done in the highest possible point groups (PGs), that is D_2h_ for **Py** and **DAP27**, and C_2h_ for **DAP16**. In the PG D_2h_, the irreducible characters of the Gaussian16 output files correspond to the standard orientation in group theory, as recommended by the International Union of Pure and Applied Chemistry (IUPAC);^35^ accordingly, the *x*-axis is oriented perpendicular to the molecular plane and the *z*-axis is passing through the 2,7 positions of the pyrene core. Singlet (S_n_) and triplet energies (T_n_) were obtained as single point TD-DFT calculations in vacuum (6-311G­(d,p) basis set) within the linear response formalism, as implemented in the Gaussian16 program package.^36^ While the TD-DFT treatment of **DAP16** (and **DAP27**) is rather straightforward, the comparison with **Py** is a delicate issue. In fact, a precise theoretical treatment of **Py** requires multireference methods,^37,38^ which is beyond the current scope, aiming at a qualitative comparison. At the TD-DFT level, the choice of the functional (and possible implicit inclusion of solvent, through the polarizable continuum model; PCM) is an intricate matter for **Py**; in fact, it is known that standard DFT functionals like B3LYP suffer to reproduce the correct state ordering of **Py**,^37,38^ while e.g., CAM-B3LYP, ωB97XD or M06–2X in vacuum give the correct ordering. However, the stabilization of S_1_ is substantially underestimated, giving Δ*E*_12_ < 0.1 eV;^37,38^ inclusion of implicit solvents may again reverse the state ordering. Thus, we consistently used M06-2X in vacuum in the current work. We note in this context that the absolute transition energies are considerably overestimated by the M06-2X functional; however, for the intended qualitative comparison of state ordering and composition between the three compounds, this functional represents the best choice. Comparison of different functionals (M06-2X, ωB97XD, PBE0, D3-B3LYP, CAM-B3LYP) in vacuum and DCM for all three compounds is found in Table S3. Furthermore, M06-2X calculated adiabatic energies for relevant excited of all compounds in vacuum and DCM are given in Table S4. The spin–orbit coupling (SOC) matrix elements were calculated between the S_1,2_ and the accepting triplet states T_n_ based on the S_0_ geometry, in vacuum and DCM, via quasi-degenerate perturbation theory, as implemented in the ORCA package,^39–41^ applying a full TD-DFT scheme (M06-2*X*/6-311G­(d,p)). Intersystem crossing (ISC) rates were then estimated via a Marcus-type expression; for details, see eqs S1 and S2; all results are given in Table S5. In order to investigate the possible deactivation paths, the minimal energy crossing points (MECPs) between different triplet and singlet states were located;^42^ for this, the geometries of all respective excited states were optimized, see Figure S15 and Tables S6–S8 for details. For the identification of the photoproducts, M06-2X in DCM was used (Tables S9–S12); for the most probable (peroxide) product, D3-BLYP calculations in DCM were done for comparison (Table S13). Furthermore, we explored the reaction path along the potential energy surface, identifying energies and equilibrium geometry of the intermediates and locating the transition state (TS); see Figure S26.

## Results and Discussion

The absorption spectrum of **DAP16** in DCM solution in the near-UV range is dominated by a vibronically well-structured absorption band with an intense apparent 0–0 band^43^ at 3.32 eV (374 nm), see Figure 1. Emission occurs without notable Stokes shift and with approximate mirror symmetry vs absorption, all reflecting the rigid nature of the molecular backbone,^44^ so that the absorption features are assigned to the S_0_ → S_1_ transition. Small deviations from mirror symmetry are ascribed to the presence of a higher electronic state (S_2_) in absorption, located about 0.14 eV above S_1_ according to the TD-DFT calculations, *vide infra*. The fluorescence quantum yield is determined as Φ_F_ = 0.34, and the lifetime is τ_F_ = 0.73 ns, see Tables 1 and S16. While fluorescence color and quantum efficiency are not very different from **Py**, the extreme shortening of the lifetime by almost 3 orders of magnitude, and the vanishing apparent Stokes shift suggests a very substantial change of the electronic nature in comparison with **Py**.

**Figure 1:**
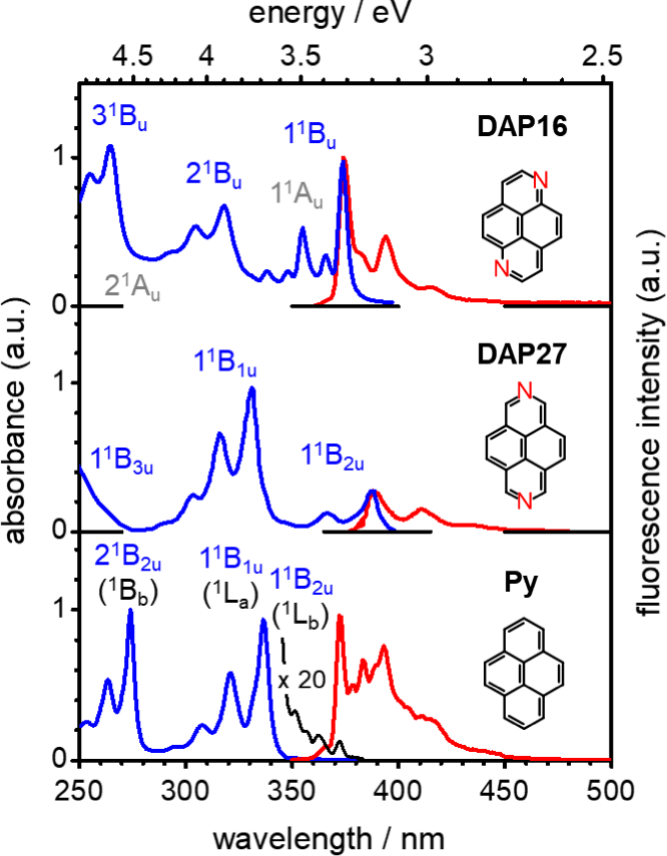
Absorption (blue) and fluorescence (red) spectra of the compounds under study. Top: **DAP16** (in DCM solution, at RT). Center: **DAP27** (in H_2_O).^27^ Bottom: **Py** (in DCM). State assignments correspond to the TD-DFT results; the low-intensity (nσ)­π* transitions (1,2^1^A_u_; in gray) for **DAP16** are estimations. The thin black line for **Py** is a 20× magnification of the low-energy absorption region.

**Table 1:** Fluorescence Quantum Yield and Lifetime (Φ_F_, τ_F_) of **DAP16** in DCM Solution (RT) in Comparison with **DAP27** and Py^a^

	Φ_F_	τ_F_/ns	*k*_r_/s^–1^	*k*_nr_/s^–1^
**DAP16**	0.34	0.73	4.7 × 10^8^	9.0 × 10^8^
**DAP27**	0.50^b^	10^b^	5.0 × 10^7^	5.0 × 10^7^
**Py**	0.65^c^	382^c^	1.7 × 10^6^	9.2 × 10^5^

a Radiative and nonradiative rates *k*_r_, *k*_nr_ are calculated via *k*_r_ = Φ_F_/τ_F_ and *k*_nr_ = (1 – Φ_F_)/τ_F_.

b In H_2_O; from ref. 27.

c In cyclohexane; from ref. 26.

It is well-known that **Py** exhibits a peculiar electronic structure,^45–47^ as reflected in the subsequent state ordering,^37,38,48–50^ vibronics,^50–54^ photokinetics^24,26^ and the ability for dynamic excimer formation.^55–57^ As seen in Figure 1, the absorption spectrum of **Py** in the near UV is dominated by the S_0_ → S_2_ transition (1^1^B_1u_; or ^1^L_a_ in the Platt notation), with an apparent 0–0 band^45^ at 3.68 eV, i.e., λ = 337 nm, and an oscillator strength *f* ≈ 0.5; see Table S3. This transition is oriented along the long molecular axis (*z*), and is described almost exclusively by a monoelectronic excitation (Φ_1_) between the highest occupied and lowest unoccupied MOs (HOMO, LUMO), here abbreviated as H → L. The subsequent excited symmetry-allowed one-electron configurations H → L + 1 and H – 1 → L (Φ_2,3_) exhibit B_2u_ symmetry and are oriented along the short axis (*y*) of **Py**. Equal energy spacing in the sets of occupied and unoccupied MOs is observed, as in fact expected for an alternant hydrocarbon; in particular Δ*E*_H,H–1_ ≈ Δ*E*_L,L+1_. This gives rise to strong first order configuration interaction (CI) between Φ_2,3_ with almost equal CI coefficients, and generates substantial splitting of the resulting (symmetry allowed) B_2u_ states. Subsequently, the lower state (1^1^B_2u_; ^1^L_b_ in the Platt notation) carries only very little oscillator strength (*f* ≈ 6 × 10^–4^),^46,58,59^ due to this alternant pairing effect. Experimentally, 1^1^B_2u_ is found as the S_1_ state (with an onset at about 3.3 eV in DCM, see Figure 1) below 1^1^B_1u_ (S_2_; onset at about 3.6 eV); the actual energy difference Δ*E*_12_ (in DCM 0.3 eV) is very sensitive to the environment as solvent shifts depend significantly on *f*.^60–63^ Within the TD-DFT framework, standard DFT functionals like B3LYP reproduce quite well the energies of bright states (i.e., with large *f*; like 1^1^B_1u_), but have difficulties to correctly govern alternant pairing (like for 1^1^B_2u_); in particular, they give the wrong state ordering for **Py** (see Table S3). On the other hand, M06-2X overestimates the absolute transition energies (by about 0.7 eV), and underestimates Δ*E*_12_, giving only 0.05 eV (in vacuum); however, it gives a good estimation of the oscillator strength (*f*_01_ = 5.7 × 10^–4^), and, importantly in the current context, the correct state ordering.^37,38^ The good performance of multiconfigurational methods for 1^1^B_2u_ of **Py** ^37,38^ points to significant second order CI contributions,^64^ which are thought to be promoted by the large exchange integral, reflected in the singlet–triplet gap of 1.5 eV between 1^1^B_1u_ and 1^3^B_1u_; see Tables S1, S2 and Figure S13 with the detailed discussion there.^65^ While S_1_ (1^1^B_2u_) is essentially dark in absorption (Figure 1), it is not dark in emission. This is due to the fact, that the very small radiative rate *k*_r_, which result from the small *f*_01_,^66^ competes well with the very small rate of nonradiative decay *k*_nr_ (Table 1); the latter proceeds both through (thermally activated) internal conversion (IC, and subsequent vibrational relaxation; VR) to S_0_, and intersystem crossing (ISC) to the triplet manifold.^67,68^ The low *k*_IC_ and *k*_ISC_ rates are due to the rigid nature of **Py**, and, at the same time, sole π­(π*) character of the relevant frontier MOs (Figure 2), so that ISC is El-Sayed forbidden, resulting in small spin–orbit coupling (SOC) for ISC. In all, the small *k*_IC_ and *k*_ISC_ gives rise to a high Φ_F_, and, at the same time, an extraordinarily long τ_F_ for **Py**; e.g., in degassed cyclohexane, Φ_F_ = 0.65, and τ_F_ = 382 ns,^24,26^ see Table 1.

**Figure 2:**
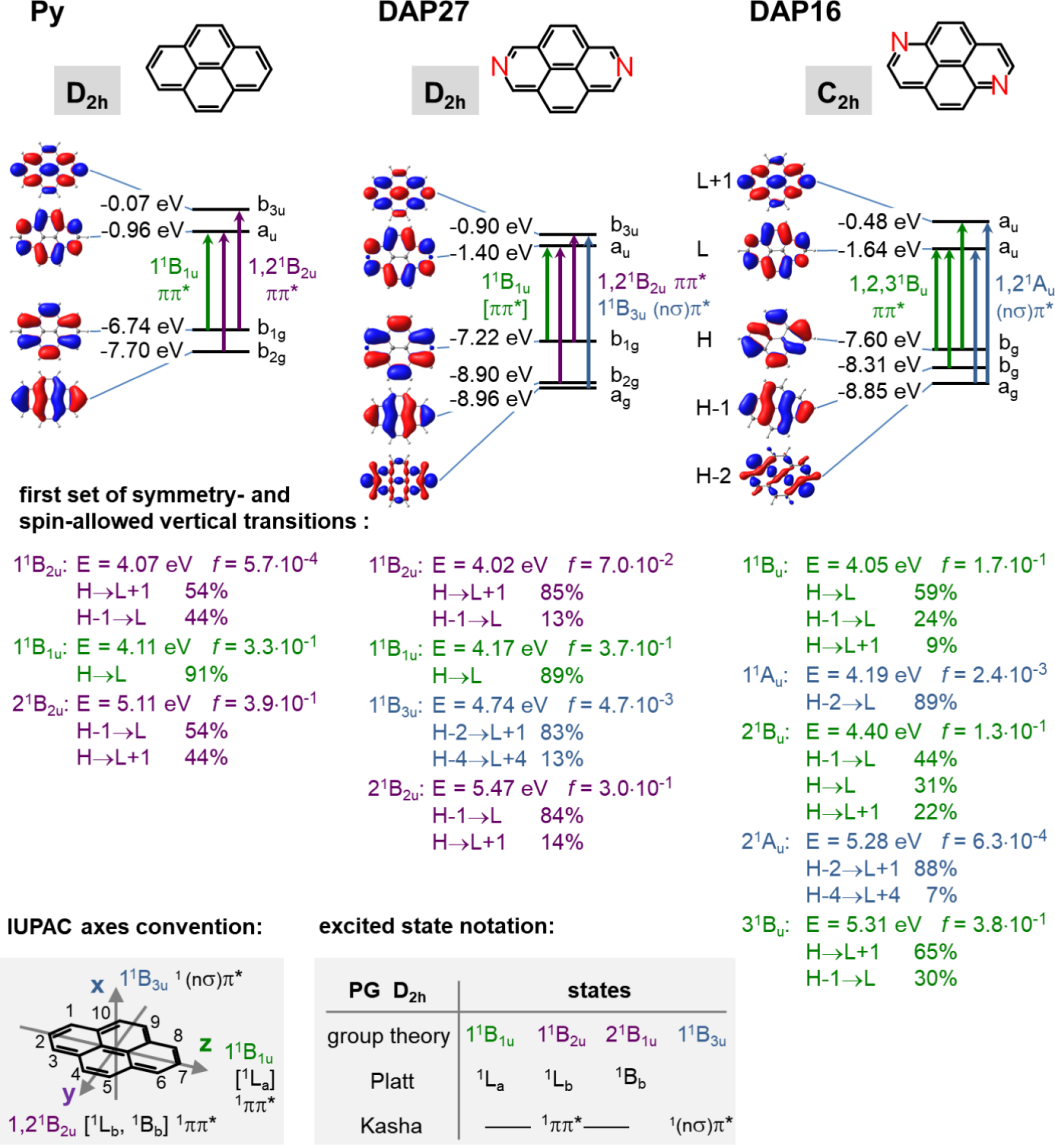
Frontier MO diagrams (energies, topologies and symmetries; H = HOMO, L = LUMO) for **Py**, **DAP27** and **DAP16**, as calculated by DFT (M06-2X, 6-311G*); below: TD-DFT calculated low-lying symmetry-allowed vertical excited state energies, oscillator strengths and MO descriptions (contributions >5%). Insets: convention for the symmetry axes in the PG D_2h_, according to IUPAC;^34^ excited state notation according to group theory, Platt^69^ and Kasha.^70^

Due to the peculiar electronic situation in **Py**, essentially all chemical modifications of the pyrene backbone break the symmetry between the sets of occupied and unoccupied MOs and modulate electronic transition energies and strengths. However, the resulting effect depends subtly on the nature, number and position of the substituents. In particular, significant differences are expected for 2,7- vs 1,6-substitution, as the H, L orbitals of **Py** exhibit a nodal plane passing through the 2,7 positions.^48,71^ Therefore, substitution at the 2,7-positions affects H – 1 and L + 1 energies much more compared to H, L. Depending on the nature of the substituents, *inter alia* substantial stabilization or destabilization of the MOs is observed, with, in most cases, enhanced *f*_01_.^48,71–73^ This is exactly what is observed for **DAP27**, where the higher electronegativity of nitrogen compared to carbon stabilizes H – 1 and L + 1 much stronger compared to, H, L, respectively, so that Δ*E*_H,H–1_ is substantially larger than Δ*E*_L,L+1_ (Figure 2). As the PG of **DAP27** is the same as **Py** (i.e., D_2h_), S_1_ is equally formed by linear combination of Φ_2,3_. However, as Δ*E*_H,H–1_ ≫ ΔE_L,L+1_, the CI coefficients are now substantially different, which strongly enhances *f*_01_ in **DAP27** in comparison with **Py**. According to our TD-DFT (M06-2X) calculations, the enhancement amounts to almost 2 orders of magnitude (with *f*_01_ = 7.0 × 10^–2^, see Figure 2). This is in good agreement with the observed spectral features of **DAP27** (Figure 1); the enhanced *f*_01_ (and, therefore, equally enlarged *k*_r_)^66^ is as well responsible for the substantial reduction of τ_F_ compared with **Py** to about 10 ns (in H_2_O), while Φ_F_ stays high (50%);^26^ see Table 1. In any case, it is noted that the appearance of the absorption spectrum of **DAP27** is still not fundamentally different from **Py**, with a dominating S_2_ band in the near UV, and a relatively weak S_1_ band, see Figure 1.

While the modulation of pyrene photophysics in **DAP27** is still moderate, **DAP16** exhibits much more drastic changes, despite the fact that the alterations in MO energies are less pronounced compared to 2,7-substitution. In fact, in **DAP16**, ΔE_H,H–1_ is now smaller than Δ*E*_L,L+1_ (see Figure 2), because the LCAO coefficients at the nitrogen atoms are smaller for H – 1 and L + 1 compared to H, L. The overall asymmetry in the energies between the occupied and unoccupied MOs is however much less pronounced compared to **DAP27**, because of the mentioned symmetry of the nodal plane in H, L. Therefore, the reason for the much more manifest spectral changes for **DAP16** is attributed to the reduction in the molecular symmetry when going from **Py** or **DAP27** (PG D_2h_) to **DAP16** with PG C_2h_. For this reason, H, and H – 1 in **DAP16** belong to the same irreducible representation (b_g_), whereas L, L + 1 belong to a_u_, see Figure 2. This mixes all three one-electron configurations Φ_1,2,3_, i.e., H → L, H – 1 → L, and H → L + 1, resulting in three optically allowed ^1^B_u_ states of similar intensities; in particular, the S_1_ state of **DAP16** carries substantial oscillator strength. The TD-DFT calculations in fact give *f*(1^1^B_u_) = 0.17, and *f*(2^1^B_u_) = 0.13, which reproduce well the observed experimental absorption features in Figure 1. The enhancement of *f*_01_ vs **Py** amounts to a factor of about 3 × 10^2^ (Figure 2); this correlates well with the enhancement of the radiative rate,^66^ see Table 1.

In contrast to **Py**, the presence of the free electron pair of nitrogen in **DAP16** generates an energetically high-lying nσ-type MO (being H–2; see Figure 2), while the corresponding σ-type MO in **Py** is very low in energy, i.e., H–6. In **DAP16** with PG C_2h_, this gives rise to a symmetry-allowed (nσ)­π*-type transition, which is found as S_2_ state (1^1^A_u_) of low oscillator strength (*f*_2_ = 2.4 × 10^–3^) between the two first ππ*-type ^1^B_u_ states, see Figure 2 and Table S1.^74^ This should somewhat broaden the absorption features in the region of the S_0_ → S_1_ transition, as indeed experimentally observed (*vide supra*). The correlated (nσ)­π*-type triplet state (T_3_; 1^3^A_u_) is calculated to be only 0.26 eV below S_1_ (see Tables S1 and S2) due to the small exchange integral for 1A_u_. This opens a new, El-Sayed allowed ISC channel in **DAP16**, which is expected to significantly enhance *k*_ISC_. This agrees with the largely increased *k*_nr_ compared to **Py**; i.e., by 3 orders of magnitude, see Table 1; TD-DFT calculations of *k*_ISC_ confirm the enhancement in a good qualitative manner when comparing the compounds; however, the calculations fail to quantitatively reproduce the effect (see Table S5a). In **DAP27**, the (nσ)­π*-type singlet and triplet states (1^1^B_3u_, 1^3^B_3u_) are significantly higher in energy compared to **DAP16** (Figure 2), so that 1^3^B_3u_ is found 0.17 eV above S_1_ (see Tables S1 and S2); therefore, *k*_nr_ (mainly via *k*_ISC_) in **DAP27** takes a position in between **Py** and **DAP16**, see Table 1, which is again qualitatively confirmed by the TD-DFT calculations, see Table S5b. The competition of radiative decay and ISC in **DAP16** results in the moderately high Φ_F_ of 34% and the short lifetime of τ_F_ = 0.73 ns; thus, τ_F_ is not only drastically shorter than in **Py**,^26^ but also considerable shorter compared to **DAP27**,^27^ see Table 1.

In the next step, we explored the deactivation paths of the triplet state. At room temperature (RT) under O_2_-free conditions, **Py** exhibits a phosphorescence lifetime of τ_P_ = 9 ms, while at low temperatures (LT) the lifetime increases to 0.5 s.^75^ In contrast, no RT phosphorescence was seen for **DAP16** and **DAP27** in Ar-purged DCM solution, pointing to very efficient nonradiative decay via triplet-singlet crossing. In fact, nonradiative pathways are known to be effectively activated when going from PAHs^76^ to their aza-counterparts; see for instance naphthalene vs quinoline.^77^ We thus calculated the triplet-singlet minimum energy crossing point (MECP) which occurs from T_2_ to the ground state S_0_ for **DAP16**, which indeed shows a local out-of-plane distortion of one of the N-containing rings, see Figure 3a and Table S7. After initial population of T_3_ (1^3^A_u_) via ISC from S_1_, the T_2_ state (2^3^B_u_) is rapidly populated by IC. From here, the MECP can be efficiently reached because of the pronounced energy gap of about 1 eV between T_1_ (1^3^B_u_) and T_2_ (see Table S2); in the framework of Fermi’s golden rule, such large gap considerably slows down IC, as it was in fact shown for azulene derivatives.^78^ This is expected to hold in the current case for IC from T_2_ to T_1_, but permits access to the MECP; according to the DFT results, the latter is in fact located 0.02 eV below the T_2_ minimum. Under LT conditions (65 K), for **DAP27** still no phosphorescence was detected, while **DAP16** does exhibit phosphorescence at 564 nm (Figure 3b), being 1.1 eV below S_1_ (reasonably reproduced by TD-DFT, see Table S3). The corresponding lifetime amounts τ_P_ = 0.29 s (Figure S17); similar to **Py**, the long τ_P_ of **DAP16** reflects the small SOC of T_1_ deactivation due to the ππ* nature of the S_0_ → T_1_ transition (Table S2). T_1_ is almost exclusively described by a H → L excitation (see Table S2), different from S_1_ (Figure 2 and Table S1), so that the natural transition orbital (NTO) topologies differ slightly in the central carbon atoms (Figure S14); this is subsequently reflected in the somewhat different vibronic patterns of the LT fluorescence and phosphorescence spectra; see Figure 3b.

**Figure 3:**
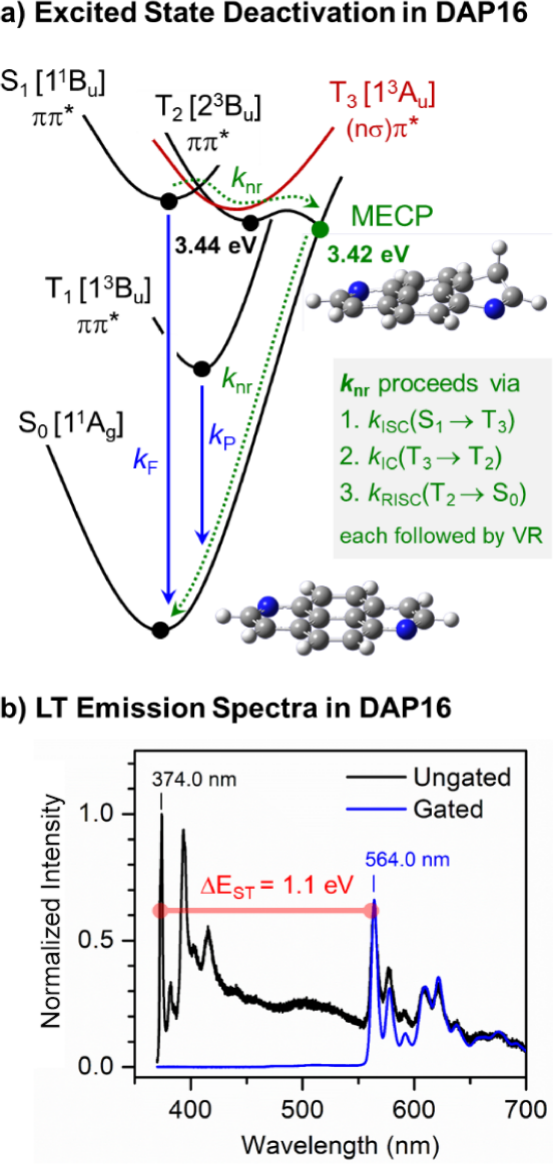
Excited state deactivation in **DAP16**. (a) Schematic path at RT (based on the experimental and computational results). Main channels from S_1_ (1^1^B_u_) are *k*_F_ and *k*_nr_ via El-Sayed allowed ISC to T_3_ (1^3^A_u_), followed by IC to T_2_ (2^3^B_u_) and reverse (R)­ISC to S_0_ via the MECP; each followed by VR. IC from T_2_ to T_1_ is a minor path (large Δ*E*); IC becomes active at LT, so that *k*_P_ opens. (TD)­DFT optimized geometries are given for S_0_ and MECP. (b) ungated (black) and gated (blue) emission spectra of **DAP16** in PMMA at LT (65 K).

The efficient population of the triplet manifold of **DAP16** (*vide supra*) allows for an intriguing photoreaction with O_2_ under unpurged conditions, while this is not observed for **DAP27**, nor for **Py** (see Figures S20 and S21). In fact, under laser irradiation at λ_ex_ = 355 nm of **DAP16** in DCM solution, new absorption features appear. The new bands resemble those of **DAP16**, but appear clearly somewhat broader and with a distinct bathochromic shift of 0.12 eV (13 nm), see Figure 4. After 20 min under the given excitation conditions, the reaction is complete, as the original **DAP16** features have entirely disappeared. The reaction does not occur under Ar-purged conditions (see Figure S22). Strikingly, the photoreaction is reversible; 7 days after the formation of photoproduct, and keeping the solution at RT in a sealed vial, the original **DAP16** spectrum slowly recovers (Figure S23). At elevated temperature (50 °C) in chloroform solution, the process is slightly accelerated (Figure S25) as all the photophysical and photochemical properties of **DAP16** are identical in DCM and chloroform (Figure S24); *a posteriori* purging with Ar does not have a notable effect.

**Figure 4:**
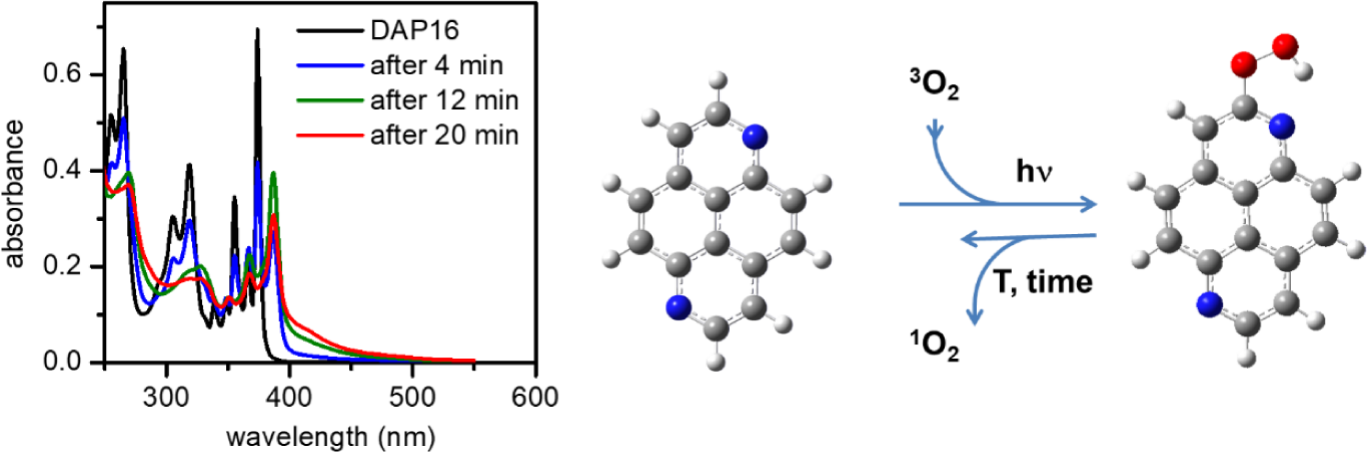
Photooxidation of **DAP16**. Absorption spectra of in air-saturated DCM solution during irradiation with a 355 nm laser (left); suggested peroxide formation (right).

We have made significant efforts to identify and isolate the photoproduct by ^1^H NMR and HRMS; however, these attempts have been unsuccessful. The solubility of O_2_ and the observed reversibility of the process may account for the lack of evidence while reproducing this phenomenon in the required 10^–2^–10^–3^ M range. Therefore, in order to gain further insight in the nature of the photoproduct, we used TD-DFT to identify the most probable candidates; in fact, TD-DFT is known to predict reliably even subtle electronic and geometrical substituent effects in conjugated compounds, for varying nature,^79–81^ number^81,82^ and position^81–83^ of the functional unit. The calculated possible photoproducts of **DAP16** included (i) noncovalent complexes in different configurations, (ii) various cycloadditions, (iii) oxidation in different positions, as well as (iv) peroxide formation after singlet oxygen production via the T_1_ state of **DAP16** during irradiation. As detailed in Tables S9–S13, the resulting UV/vis absorption spectra of the photoproducts turned out to be largely different for the various reactions, so that comparison with the experimental spectrum of the photoproduct allows to specifically identify the most probable species formed. Taking together the experimental findings (that is the small bathochromic shift of the absorption against **DAP16** while maintaining the spectral features, as well as the very slow back-reaction of photo-oxidation), the most probable scenario for the photoproduct is peroxide formation via insertion in the C–H bond at the 2-position of **DAP16**, see Figure 4 and Table S12. This is plausible, as the α-position to the nitrogen is expected to be activated,^84^ and the photoproduct is additionally stabilized by intramolecular H-bonding with the N atom. In fact, the N···H distance is calculated to be 1.84 Å, matching typical values for NH hydrogen bonding;^85^ for additional information on the calculated reaction path, see Figure S26. To further elucidate the photochemical process, derivatives of **DAP16**, including nitrogen-alkylated forms and N-oxides, are currently being synthesized to explore their reactivity and mechanistic pathways.

In conclusion, we synthesized the 1,6-diazapyrene isomer, **DAP16**, which has not been reported so far, and which is a well-defined small size prototype system for nitrogen-containing PAHs. The new compound dramatically changes the optical excitation, and subsequent photophysics and -chemistry compared to pyrene and the well-studied 2,7-diazapyrene; in particular, effective triplet population was observed for **DAP16**. All changes could be rationalized by the peculiarities of **DAP16** in the electronic structure and symmetry, as fully revealed by (TD)­DFT calculations on excited state properties and deactivation. Because of its unique photophysics, **DAP16** shows an extreme sensitivity against oxygen, leading to reversible peroxide formation. The extraordinary sensitivity of positional isomerism in diazapyenes for photophysics and -chemistry is considered of high relevance for a general understanding of N-PAHs and their targeted design.

## Supplementary Material



## Data Availability

All detailed experimental and characterization data associated with this work are available in the Supporting Information, raw data are available at Zenodo repository: 10.5281/zenodo.14277234.

## Notes

Detailed synthesis and characterization of all compounds, NMR and HRMS copy, symmetry considerations for pyrene, additional calculations, spectroscopic results and photochemical experiments (PDF)

## References

[1] Stępień M., Gońka E., Żyła M., Sprutta N. (2017). Heterocyclic Nanographenes
and Other Polycyclic Heteroaromatic Compounds: Synthetic Routes, Properties,
and Applications. Chem. Rev..

[2] Hu C., Li M., Qiu J., Sun Y. P. (2019). Design and fabrication of carbon
dots for energy conversion and storage. Chem.
Soc. Rev..

[3] Kotwica K., Wielgus I., Proń A. (2021). Azaacenes Based Electroactive Materials:
Preparation, Structure, Electrochemistry, Spectroscopy and Applications—A
Critical Review. Materials.

[4] Shao X., Aquino A. J., Otyepka M., Nachtigallová D., Lischka H. (2020). Tuning the UV spectrum of PAHs by
means of different
N-doping types taking pyrene as paradigmatic example: Categorization
via valence bond theory and high-level computational approaches. Phys. Chem. Chem. Phys..

[5] Borissov A., Maurya Y. K., Moshniaha L., Wong W. S., Żyła-Karwowska M., Stepien M. (2022). Recent advances in heterocyclic nanographenes and other
polycyclic heteroaromatic compounds. Chem. Rev..

[6] Tian X., Shoyama K., Würthner F. (2023). Nitrogen-doped polycyclic aromatic
hydrocarbons by a one-pot Suzuki coupling/intramolecular SN Ar reaction. Chem. Sci..

[7] Tucker S. A., Darmodjo H., Acree W. E., Zander M., Meister E. C., Tanga M. J., Tokita S. (1992). Polycyclic Aromatic Nitrogen Heterocycles.
Part IV: Effect of Solvent Polarity, Solvent Acidity, Nitromethane
and 1, 2, 4-Trimethoxybenzene on the Fluorescence Emission Behavior
of Select Monoaza-and Diazaarenes. Appl. Spectrosc..

[8] Müller M., Ahrens L., Brosius V., Freudenberg J., Bunz U. H. (2019). Unusual stabilization of larger acenes and heteroacenes. J. Mater. Chem. C.

[9] Chen F., Melle-Franco M., Mateo-Alonso A. (2022). Planar and Helical Dinaphthophenazines. J. Org. Chem..

[10] Molenda R., Boldt S., Villinger A., Ehlers P., Langer P. (2020). Synthesis
of 2-Azapyrenes and Their Photophysical and Electrochemical Properties. J. Org. Chem..

[11] Vardanyan A., Boldt S., Villinger A., Ehlers P., Langer P. (2022). Synthesis
and Properties of 1-Azapyrenes. J. Org. Chem..

[12] Østergaard M. E., Hrdlicka P. J. (2011). Pyrene-functionalized oligonucleotides and locked nucleic
acids (LNAs): Tools for fundamental research, diagnostics, and nanotechnology. Chem. Soc. Rev..

[13] Bains G., Patel A. B., Narayanaswami V. (2011). Pyrene: A
probe to study protein
conformation and conformational changes. Molecules.

[14] Figueira-Duarte T. M., Mullen K. (2011). Pyrene-based materials for organic electronics. Chem. Rev..

[15] Feng X., Hu J.-Y., Redshaw C., Yamato T. (2016). Functionalization of
pyrene to prepare luminescent materials—typical examples of
synthetic methodology. Chem. - Eur. J..

[16] Kinik F. P., Ortega-Guerrero A., Ongari D., Ireland C. P., Smit B. (2021). Pyrene-based
metal organic frameworks: From synthesis to applications. Chem. Soc. Rev..

[17] Kowser Z., Rayhan U., Akther T., Redshaw C., Yamato T. (2021). A brief review
on novel pyrene based fluorometric and colorimetric chemosensors for
the detection of Cu^2+^. Mater. Chem.
Front..

[18] Borovlev I. V., Demidov O. P. (2003). Diazapyrenes. Chem. Heterocycl.
Compd..

[19] Taniya O. S., Khasanov A. F., Varaksin M. V., Starnovskaya E. S., Krinochkin A. P., Savchuk M. I., Kopchuk D. S., Kovalev I. S., Kim G. A., Nosova E. V. (2021). Azapyrene-based
fluorophores:
Synthesis and photophysical properties. New
J. Chem..

[20] Zhirov A.
M., Kovalev D. A., Ulshina D. V., Pisarenko S. V., Demidov O. P., Borovlev I. V. (2020). Diazapyrenes:
Interaction with nucleic
acids and biological activity. Chem. Heterocycle.
Compd..

[21] Mukherjee A., Akulov A. A., Santra S., Varaksin M. V., Kim G. A., Kopchuk D. S., Taniya O. S., Zyryanov G. V., Chupakhin O. N. (2022). 2, 7-Diazapyrenes:
A brief review on synthetic strategies and application opportunities. RSC Adv..

[22] Becker H. C., Nordén B. (1997). DNA binding
properties of 2, 7-diazapyrene and its
N-methylated cations studied by linear and circular dichroism spectroscopy
and calorimetry. J. Am. Chem. Soc..

[23] Sun J., Liu Z., Liu W. G., Wu Y., Wang Y., Barnes J. C., Hermann K. R., Goddard W. A., Wasielewski M. R., Stoddart J. F. (2017). Mechanical-bond-protected,
air-stable radicals. J. Am. Chem. Soc..

[24] Ramírez-Barroso S., Jacobo-Martín A., Navarro-Baena I., Hernández J. J., Navio C., Rodríguez I., Wannemacher R. (2021). On the nature
of solvothermally synthesized carbon nanodots. J. Mater. Chem. C.

[25] Birks J. B., Dyson D. J., Munro I. H. (1963). ’Excimer’
fluorescence
II. Lifetime studies of pyrene solutions. Proc.
R. Soc. London, Ser. A.

[26] Karpovich D. S., Blanchard G. J. (1995). Relating the polarity-dependent fluorescence response
of pyrene to vibronic coupling. Achieving a fundamental understanding
of the py polarity scale. J. Phys. Chem..

[27] Becker H. C., Broo A., Nordén B. (1997). Ground-and excited-state properties
of molecular complexes between adenine and 2, 7-diazapyrene and its
N-methylated cations. J. Phys. Chem. A.

[28] Dobereiner G. E., Crabtree R. H. (2010). Dehydrogenation
as a substrate-activating strategy
in homogeneous transition-metal catalysis. Chem.
Rev..

[29] Gunanathan C., Milstein D. (2013). Applications of acceptorless dehydrogenation and related
transformations in chemical synthesis. Science.

[30] Llabres-Campaner P. J., Ballesteros-Garrido R., Ballesteros R., Abarca B. (2018). Straight access to
indoles from anilines and ethylene glycol by heterogeneous acceptorless
dehydrogenative condensation. J. Org. Chem..

[31] Bellezza D., Zaragozá R. J., José Aurell M., Ballesteros R., Ballesteros-Garrido R. (2021). Acceptorless dehydrogenative condensation: Synthesis
of indoles and quinolines from diols and anilines. Org. Biomol. Chem..

[32] Gonzalez-Sanchis N., Perez-Quilez P., Bellezza D., Flor-Sanchez A., Ballesteros R., Ballesteros-Garrido R. (2022). Polycyclic Aromatic N-Heterocycles
(PANHs) from Naphthyl and Anthracenyl Amines and Diols. Synthesis.

[33] Sotiriou-Leventis C., Mao Z. (2000). A facile synthesis
of 2, 7-diazapyrene. J.
Heterocycl. Chem..

[34] Würth C., Grabolle M., Pauli J., Spieles M., Resch-Genger U. (2011). Comparison
of methods and achievable uncertainties for the relative and absolute
measurement of photoluminescence quantum yields. Anal. Chem..

[35] Mulliken R. S. (1955). Electronic
population analysis on LCAO–MO molecular wave functions. I. J. Chem. Phys..

[36] Frisch, M. J. ; Trucks, G. W. ; Schlegel, H. B. ; Scuseria, G. E. ; Robb, M. A. ; Cheeseman, J. R. ; Scalmani, G. ; Barone, V. ; Petersson, G. A. ; Nakatsuji, H. , Gaussian 16, Revision C.01, Gaussian Inc., 2016.

[37] Shi B., Nachtigallová D., Aquino A. J. A., Machado F. B. C., Lischka H. (2019). High-level
theoretical benchmark investigations of the UV-vis absorption spectra
of paradigmatic polycyclic aromatic hydrocarbons as models for graphene
quantum dots. J. Chem. Phys..

[38] Shirai S., Inagaki S. (2020). Ab initio study on the excited states of pyrene and
its derivatives using multi-reference perturbation theory methods. RSC Adv..

[39] Neese F. (2012). The ORCA program
system. Wiley Interdiscip. Rev.: Comput. Mol.
Sci..

[40] Neese F. (2018). Software update:
The ORCA program system, version 4.0. Wiley
Interdiscip. Rev.: Comput. Mol. Sci..

[41] De
Souza B., Farias G., Neese F., Izsak R. (2019). Predicting
phosphorescence rates of light organic molecules using time-dependent
density functional theory and the path integral approach to dynamics. J. Chem. Theory Comput..

[42] Lu, T. sobMECP program, http://sobereva.com/286 (Accessed 11 November 2024).

[43] Heimel G., Daghofer M., Gierschner J., List E. J. W., Grimsdale A. C., Müllen K., Beljonne D., Brédas J.-L., Zojer E. (2005). Breakdown of the mirror
image symmetry in the optical absorption/emission
spectra of oligo (para-phenylene)­s. J. Chem.
Phys..

[44] Gierschner J., Mack H. G., Lüer L., Oelkrug D. (2002). Fluorescence and absorption spectra of oligophenylenevinylenes:
Vibronic coupling, band shapes, and solvatochromism. J. Chem. Phys..

[45] Platt J. R. (1954). The box
model and electron densities in conjugated systems. J. Chem. Phys..

[46] Becker R. S., Singh I. S., Jackson E. A. (1963). Comprehensive
Spectroscopic Investigation
of Polynuclear Aromatic Hydrocarbons. I. Absorption Spectra and State
Assignments for the Tetracyclic Hydrocarbons and their Alkyl-Substituted
Derivatives. J. Chem. Phys..

[47] Thulstrup E. W., Downing J. W., Michl J. (1977). Excited singlet
states of pyrene.
Polarization directions and magnetic circular dichroism of azapyrenes. Chem. Phys..

[48] Crawford A. G., Dwyer A. D., Liu Z., Steffen A., Beeby A., Palsson L. O., Tozer D. J., Marder T. B. (2011). Experimental
and
theoretical studies of the photophysical properties of 2-and 2, 7-functionalized
pyrene derivatives. J. Am. Chem. Soc..

[49] Graef E. L., Martins J. B. L. (2019). Analysis of lowest
energy transitions at TD-DFT of
pyrene in vacuum and solvent. J. Mol. Model.

[50] Chadwick R. J., Wickham K., Besley N. A. (2020). Simulation of vibrationally
resolved
absorption spectra of neutral and cationic polyaromatic hydrocarbons. Theor. Chem. Acc..

[51] Mangle E. A., Topp M. R. (1986). Excited-state dynamics
of jet-cooled pyrene and some
molecular complexes. J. Phys. Chem..

[52] Freidzon A. Y., Valiev R. R., Berezhnoy A. A. (2014). Ab initio
simulation of pyrene spectra
in water matrices. RSC Adv..

[53] D’Abramo M., Aschi M., Amadei A. (2015). Theoretical
calculation of the pyrene
emission properties in different solvents. Chem.
Phys. Lett..

[54] Braun G., Borges I., Aquino A. J. A., Lischka H., Plasser F., Do Monte S. A., Ventura E., Mukherjee S., Barbatti M. (2022). Non-Kasha fluorescence of pyrene emerges from a dynamic
equilibrium between excited states. J. Chem.
Phys..

[55] Hoche J., Schmitt H. C., Humeniuk A., Fischer I., Mitrić R., Röhr M. I. (2017). The mechanism
of excimer formation: An experimental
and theoretical study on the pyrene dimer. Phys.
Chem. Chem. Phys..

[56] Reiter S., Roos M. K., de Vivie-Riedle R. (2019). Excited state
conformations of bridged
and unbridged pyrene excimers. ChemPhotoChem.

[57] Martínez-Abadía M., Varghese S., Gierschner J., Giménez R., Ros M. B. (2022). Luminescent assemblies of pyrene-containing bent-core
mesogens: Liquid crystals, π-gels and nanotubes. J. Mater. Chem. C.

[58] Ferguson J., Reeves L. W., Schneider W. G. (1957). Vapor absorption
spectra and oscillator
strengths of naphthalene, anthracene, and pyrene. Can. J. Chem..

[59] Ref. 46 gives molar extinction coefficients of S_1_ and S_2_, while ref. 58 gives *f* _02_; comparison allows the estimation of *f* _01_ to ca. 6 × 10^–4^.

[60] Analysis of the jet-cooled fluorescence excitation spectrum gives ΔE_12_ = 0.491 eV, see ref. 51. Analysis of the vapor absorption spectrum gave 0.435 eV and in iso-octane solution 0.381 eV was reported, see ref. 46. In the more polar DCM solution, we find about 0.30 eV. This strong solvent dependency can be understood in the framework of the Onsager model (see ref. 61) because of the much larger *f* of S_2_ (1^1^B_1u_) in comparison to S_1_ (1^1^B_2u_), i.e. by 3 orders of magnitude; for prototypical solvent shifts of bright and dark states see e.g. refs. 62 and 63.

[61] Onsager L. (1936). Electric moments
of molecules in liquids. J. Am. Chem. Soc..

[62] Kohler B. E., Itoh T. (1988). Fluorescence from the 1^1^Bu state of diphenylhexatriene:
Inversion of the 11Bu and 21Ag levels in carbon disulfide. J. Phys. Chem..

[63] Egelhaaf H. J., Gierschner J., Oelkrug D. (2002). Polarizability effects and energy
transfer in quinquethiophene doped bithiophene and OPV films. Synth. Met.

[64] Roldao J. C., Oliveira E. F., Milián-Medina B., Gierschner J., Roca-Sanjuán D. (2022). Accurate Calculation of Excited-State
Absorption for
Small-to-Medium-Sized Conjugated Oligomers: Multiconfigurational Treatment
vs Quadratic Response TD-DFT. J. Chem. Theory
Comput..

[65] Double (or higher order) excitations can be significant for optical transitions, enabled through simultaneous triplet excitations; a prominent example is the 2^1^A_g_ state in oligoenes, where double excitation is specifically accomplished by the very large exchange integral for 1B_u_ in polyenes. For a recent discussion on the relevance in other conjugated oligomers, see ref. 64. In pyrene, some double excitation contribution to 1^1^B_2u_ can be promoted by simultaneous excitation of 1^3^B_1u_ and 1^3^B_3g_ (see Figure S13), due to the large exchange integral for 1B_1u_, resulting in ΔE(1^1^B_1u_ −1^3^B_1u_) = 1.54 eV, see Tables S1, S2; for further detailed discussion, see Section 2 of the Supporting Information and Figure S13.

[66] Strickler S. J., Berg R. A. (1962). Relationship between
absorption intensity and fluorescence lifetime of molecules. J. Chem. Phys..

[67] Kropp J. L., Dawson W. R., Windsor M. W. (1969). Radiative and radiationless processes
in aromatic molecules. Pyrene. J. Phys. Chem..

[68] Hirano H., Azumi T. (1982). Temperature dependence of intersystem crossing of pyrene. Chem. Phys. Lett..

[69] Platt J. R. (1949). Classification
of spectra of cata-condensed hydrocarbons. J.
Chem. Phys..

[70] Kasha M. (1950). Characterization
of electronic transitions in complex molecules. Discuss. Faraday Soc..

[71] Merz J., Fink J., Friedrich A., Krummenacher I., Al Mamari H. H., Lorenzen S., Haehnel M., Eichhorn A., Moos M., Holzapfel M. (2017). Pyrene molecular orbital
shuffle—controlling excited state and redox properties by changing
the nature of the frontier orbitals. Chem. -
Eur. J..

[72] Qiao Y., Zhang J., Xu W., Zhu D. (2011). Novel 2, 7-substituted
pyrene derivatives: Syntheses, solid-state structures, and properties. Tetrahedron.

[73] In the presence of strong donor or acceptor 2,7-substituents, ordering of the frontier MOs may change, giving rise to low-lying charge-transfer (CT) states in some cases, see ref.^48^

[74] It is noted that theoretical calculations predict two diazapyrenes isomers, where the (nσ)π* transition appears as S_1_, being 1,2- and 4,5-diazapyrene, see ref. 4 This is easy to understand as these are the only isomers which bear an azo-unit in the core; azo-compounds are well known to generate low-lying nπ*-states, as reviewed e.g. Zollinger, H., *”Azo and Diazo Chemistry”*; Interscience Publishers, Inc.: New York, N. Y., **1961,** and in Crespi, S.; Simeth, N. A.; König, B. Heteroaryl azo dyes as molecular photoswitches. *Nat. Rev. Chem.* **2019,** *3(3),* 133–146.

[75] Langelaar J., Rettschnick R. P. H., Hoijtink G. J. (1971). Studies on triplet radiative lifetimes,
phosphorescence, and delayed fluorescence yields of aromatic hydrocarbons
in liquid solutions. J. Chem. Phys..

[76] Harabuchi Y., Taketsugu T., Maeda S. (2015). Exploration of minimum energy conical
intersection structures of small polycyclic aromatic hydrocarbons:
Toward an understanding of the size dependence of fluorescence quantum
yields. Phys. Chem. Chem. Phys..

[77] Harabuchi Y., Saita K., Maeda S. (2018). Exploring radiative and nonradiative
decay paths in indole, isoindole, quinoline, and isoquinoline. Photochem. Photobiol. Sci..

[78] Murata S., Iwanaga C., Toda T., Kokubun H. (1972). Fluorescence Yields
of Azulene Derivatives. Chem. Phys. Lett..

[79] Sancho-García J. C., Brédas J. L., Beljonne D., Cornil J., Martínez-Álvarez R., Hanack M., Poulsen L., Gierschner J., Mack H. G., Egelhaaf H. J., Oelkrug D. (2005). Design of π-conjugated
organic materials for one-dimensional energy transport in nanochannels. J. Phys. Chem. B.

[80] Milián-Medina B., Beljonne D., Egelhaaf H.-J., Gierschner J. (2007). Effect of
fluorination on the electronic structure and optical excitations of
π-conjugated molecules. J. Chem. Phys..

[81] Milian-Medina B., Anthony J. E., Gierschner J. (2008). Independent Tuning of Electronic
Levels in Pentacene by Site-Specific Substitution. ChemPhysChem.

[82] Dänekamp B., Kobin B., Bhattacharyya S., Hecht S., Milián-Medina B., Gierschner J. (2016). Tuning of
the electronic and photophysical properties
of ladder-type quaterphenyl by selective methylene-bridge fluorination. Phys. Chem. Chem. Phys..

[83] Shi J., Aguilar Suarez L. E., Yoon S.-J., Varghese S., Serpa C., Park S. Y., Luer L., Roca-Sanjuán D., Milián-Medina B., Gierschner J. (2017). Solid state luminescence enhancement
in π-conjugated materials: Unraveling the mechanism beyond the
framework of AIE/AIEE. J. Phys. Chem. C.

[84] Cornelisse J., Havinga E. (1975). Photosubstitution reactions
of aromatic compounds. Chem. Rev..

[85] Jeffrey, G. A. An Introduction to Hydrogen Bonding; Oxford University Press, 1997.

