# Supplementary material for: 1,6-Diazapyrene: A Novel, Well-Defined, Small-Size Prototype System for Nitrogen-Containing PAHs
Source: J Phys Chem A. 2025 May 12;129(20):4471–9. doi: 10.1021/acs.jpca.5c01474 (PMC12128021; doi:10.1021/acs.jpca.5c01474)
Supplement: Supplementary file 1 [file jp5c01474_si_001.pdf]

Supporting Information

1,6-Diazapyrene: A Novel Well-Defined Small-Size Prototype System for Nitrogen-containing PAHs

Indranil Bhattacharjee,[1,a] Liangxuan Wang,[1,2,a] Nerea Gonzalez-Sanchis,[3,a] Begoña Milián-Medina,[4] Rafael Ballesteros,[3] Reinhold Wannemacher,[1,*] Rafael Ballesteros-Garrido,[3,*] Johannes Gierschner[1,*]

[1] Madrid Institute for Advanced Studies, IMDEA Nanoscience, C/ Faraday 9, Ciudad Universitaria de Cantoblanco, 28049 Madrid, Spain

[2] Institute of Physical and Theoretical Chemistry, Eberhard Karls University Tübingen, 72076 Tübingen, Germany

[3] Department for Organic Chemistry, Faculty of Chemistry, University of Valencia, 46100 Burjassot, Valencia, Spain

[4] Department for Physical Chemistry, Faculty of Chemistry, University of Valencia, 46100 Burjassot, Valencia, Spain

[a] These authors have contributed equally to the work.

* e-mail: reinhold.wannemacher@imdea.org, rafael.ballesteros-garrido@uv.es, johannes.gierschner@imdea.org

Table of Contents

	page
1. Synthesis and Characterization of DAP16	2
2. Symmetry Considerations for Pyrene (PG D2h)	9
3. Additional Computational Results	10
3. Additional Spectroscopic Results	18
5. Photochemistry of DAP16	20

1. Synthesis and Characterization of DAP16

All reagents were purchased from commercially available reagents of commercial firms such as Sigma Aldrich, VWR or Scharlab, they were used without any previous treatment. ZnO was purchased as a 20 wt. % nanoparticles suspension in H_2O, <100 nm particle size ≤40 nm avg. part. size (APS), The reactions were carried out inside glass reaction tubes sealed with an Easy-On PTFE cap that were put into a Carousel 12 Plus reaction station at the desired temperatures. All reaction mixtures were filtered through a 0.45 µm PTFE 25 mm syringe filter. Silica gel plates Merck 5554 were used for thin layer chromatography during column chromatography purification. Silica from Merck 60 (0.040−0.063 mm) was employed for column chromatography. NMR spectra were recorded in a Bruker Avance 300 DPX at a frequency of 300 MHz for ^{1}H and 75 MHz for ^{13}C. Spectra were measured in deuterated chloroform ($CDCl_3$) or deuterated dimethylsulphoxide (DMSO-d6) and the chemical shifts (δ) were indicated in ppm. All spectra were referenced based on the residual signal of the solvent: 7.26 ppm for ^{1}H NMR and 77.16 ppm for ^{13}C NMR in $CDCl_3$; 2.5 ppm for ^{1}H NMR and 39.5 ppm for ^{13}C NMR in DMSO-d6. Coupling constants (J) are expressed in Hz. Melting points were measured in a Reichert-Jung melting point-microscope (Swedish). Mass spectra of high resolution (HRMS) were measured in an AB SCIEX Triple TOF mass spectrometer with an electrospray ionization (ESI) source with a capillary voltage of 4.5 kV

Naphthalene-1,5-diamine (0.21 g, 1 mmol), 1.7 % of Pt/Al_2O_3 (138 mg) and 4.5 % of ZnO dispersion (21.8 µL) were mixed in 5 mL of the ethylene glycol in a 50 mL glass reaction tube. The tube was sealed and put into a reaction station with stirring at 175°C for 44 h. The reaction mixture was cooled to room temperature. The reaction mixture was poured into water (30 mL), precipitating a black solid. Filter and wash with water. The black solid was then dissolved with ethyl acetate (50 mL) and filtered to recover the catalyst. The organic layer was washed with brine (3 × 20 mL), and then it was dried with Na_2SO_4, filtered, and concentrated under vacuum. The residue was treated with chloroform to solubilize the benzo[*lmn*][2,7]phenantroline (**DAP16**) and precipitating the pure dihydroindolo[7,6-g]indole (**BI[6,7-*g*]**). **DAP16** was purified by chromatography using silica gel and a mixture hexane/ethyl acetate as eluent from 8/2 to 5/5 vol/vol. Ultrapure samples of DAP16 were also obtained by sublimation after removal of the side product by precipitation.

1.1. Benzo[*lmn*][2,7]phenantroline (DAP16)

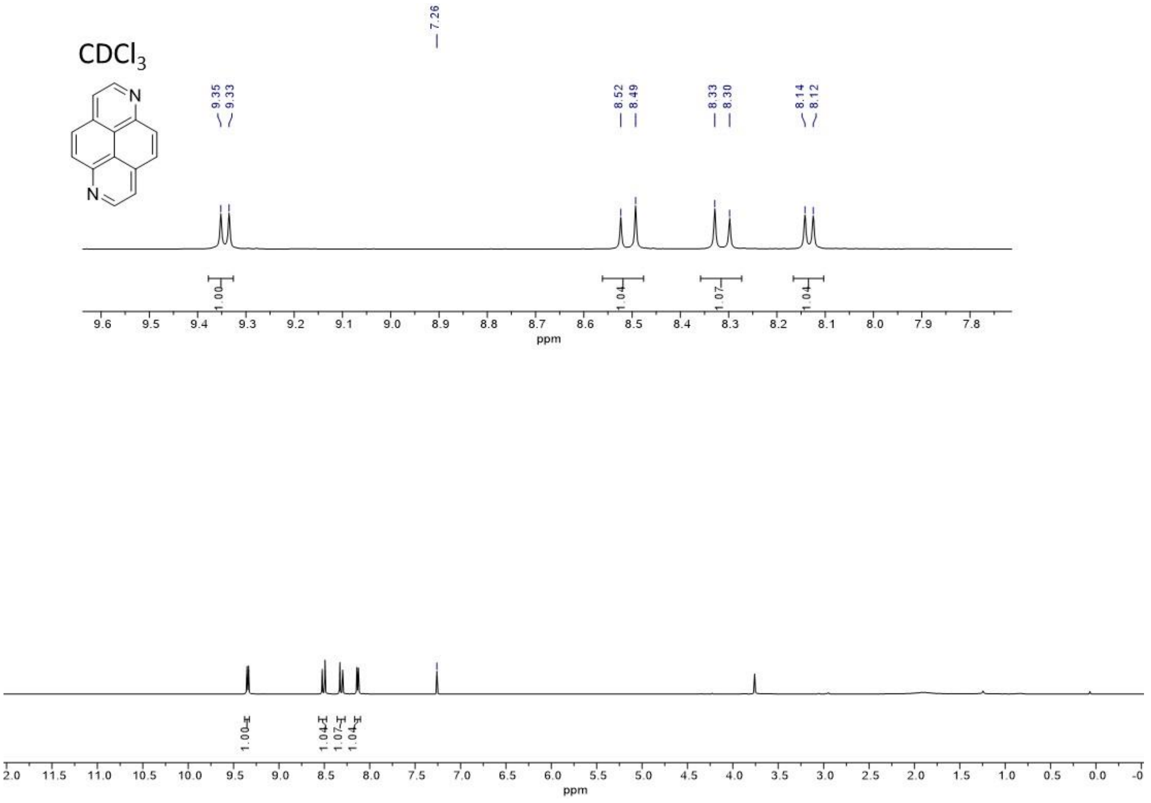

Figure S1: ^{1}H-NMR of **DAP16**

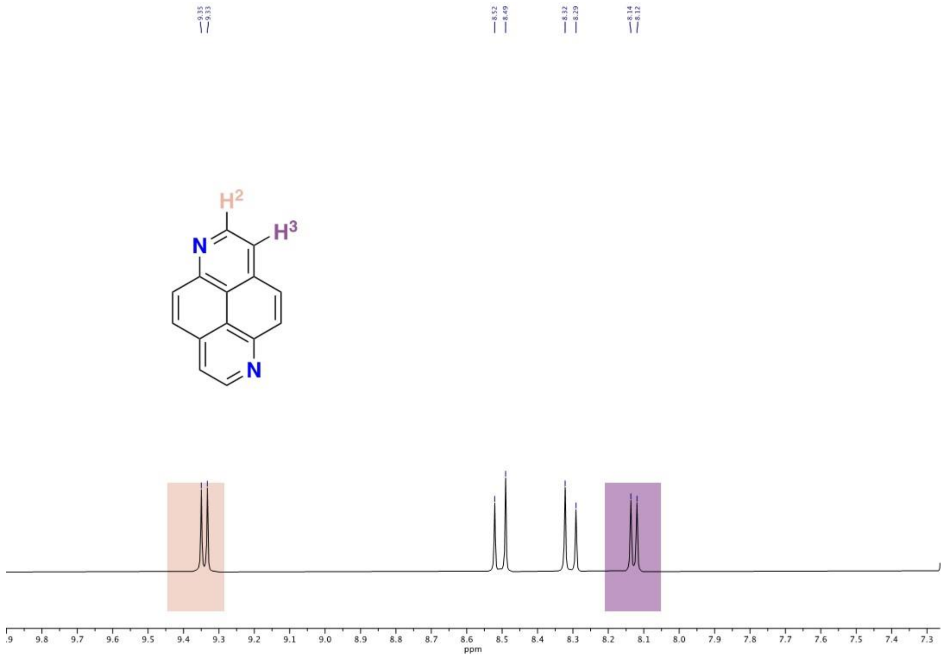

Figure S2: ^{1}H-NMR of **DAP16**, aromatic domain, signal assignation.

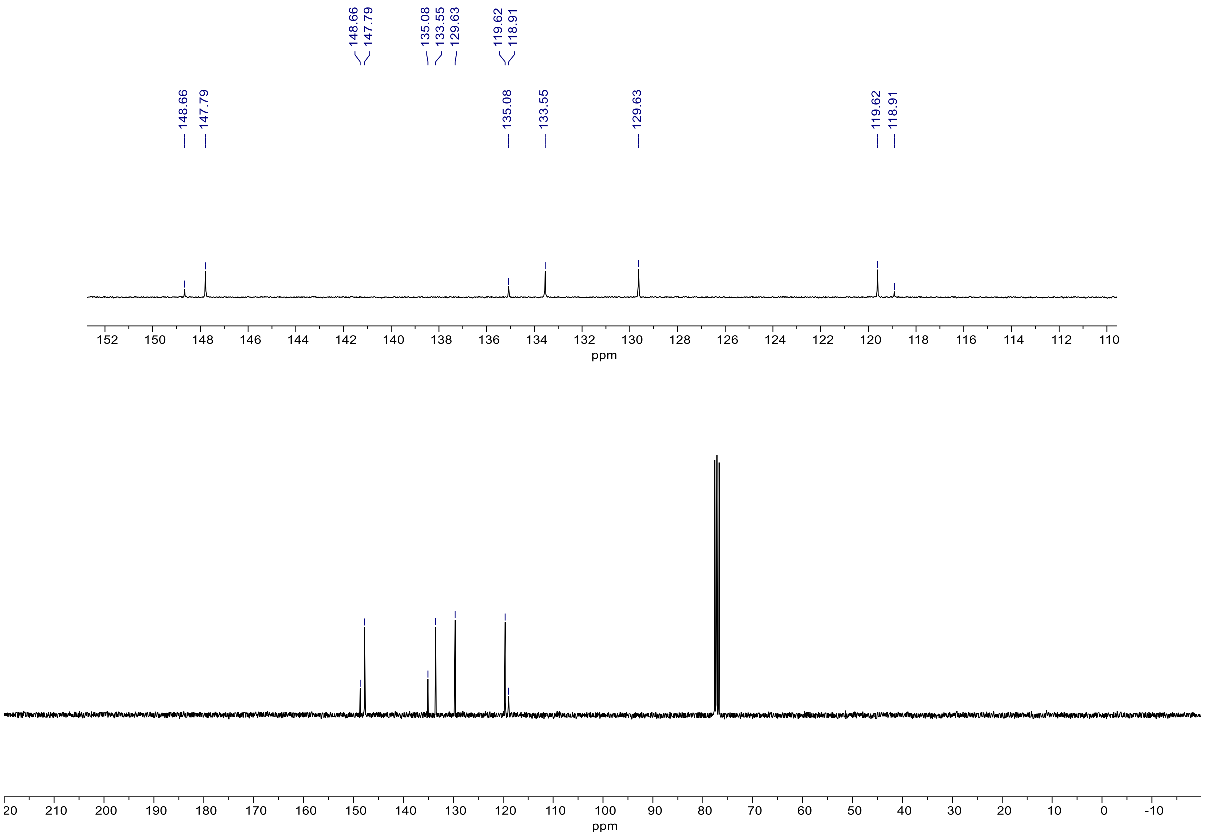

Figure S3: ^{13}C-NMR of **DAP16**.

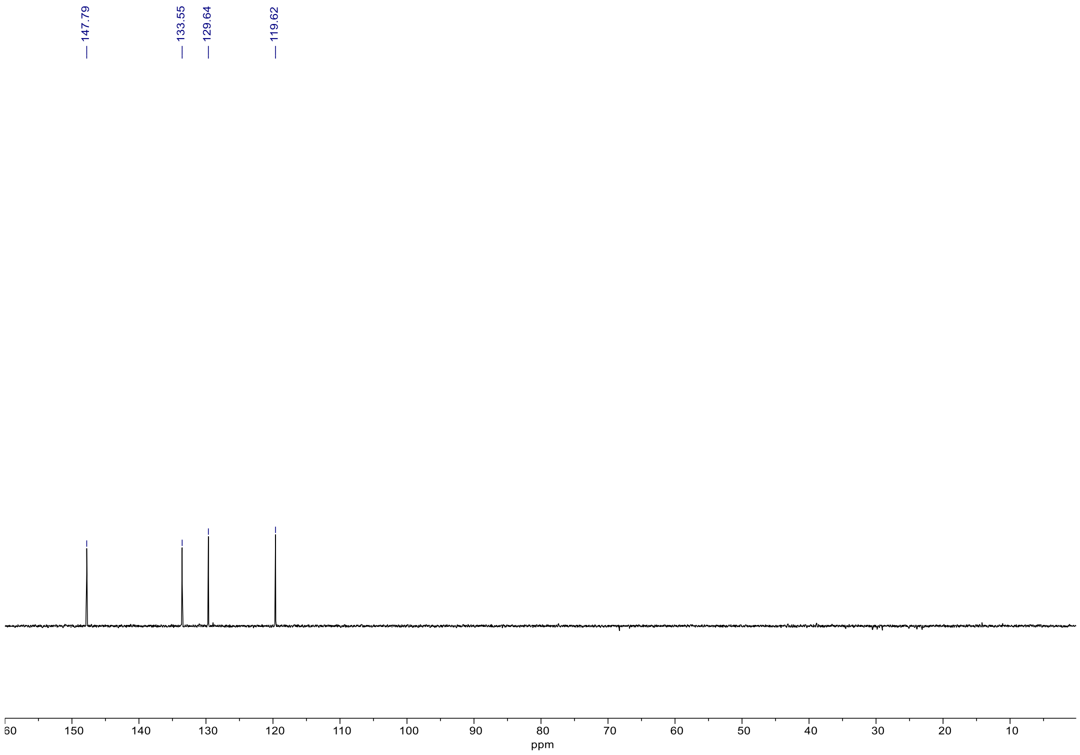

Figure S4: ^{13}C-DEPT135-NMR of **DAP16**.

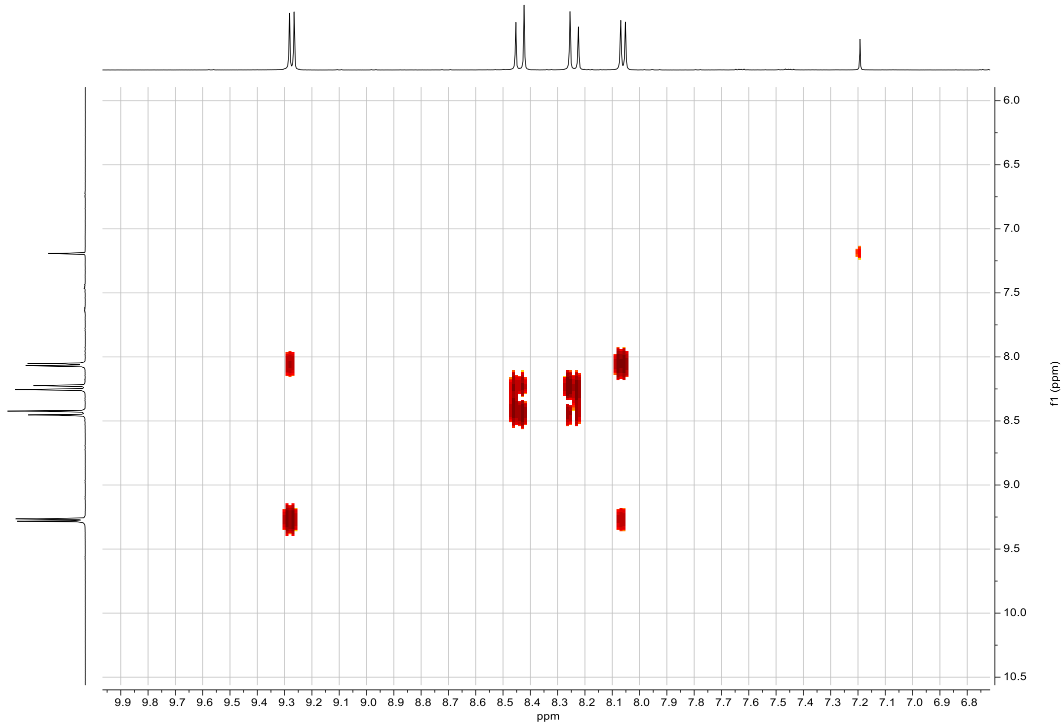

Figure S5: HH COSY-NMR of **DAP16**.

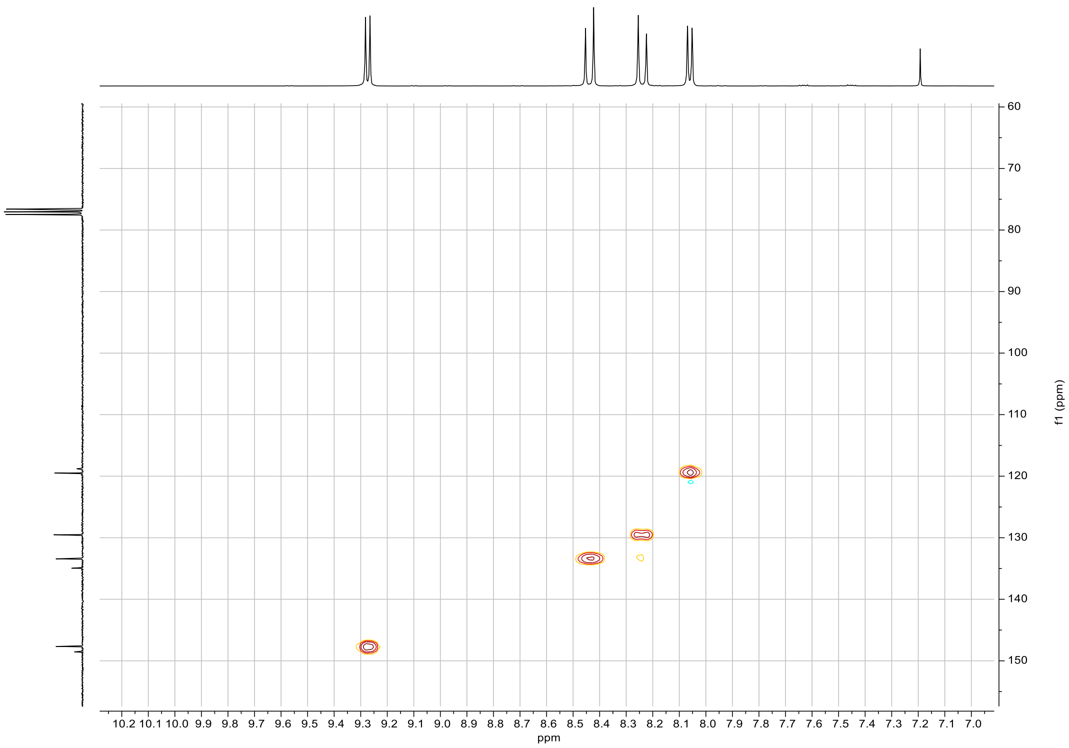

Figure S6: HSQC NMR of **DAP16**.

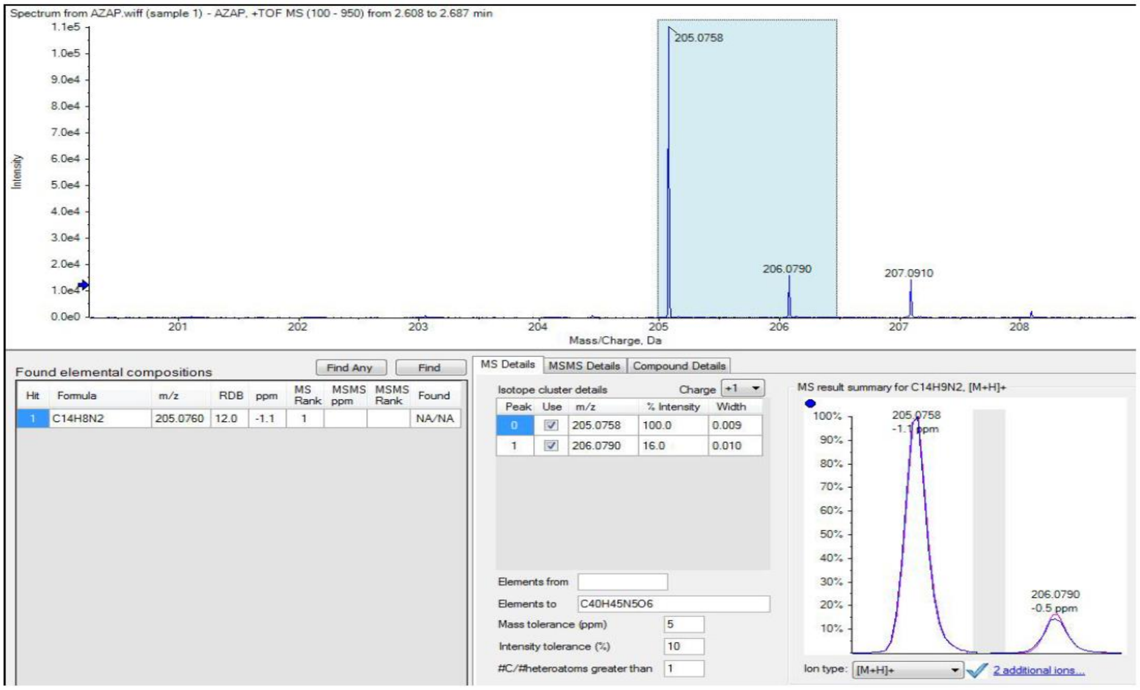

Figure S7: HRMS of **DAP16**.

1.2. Dihydroindolo[7,6-*g*]indole (BI[6,7-*g*]).

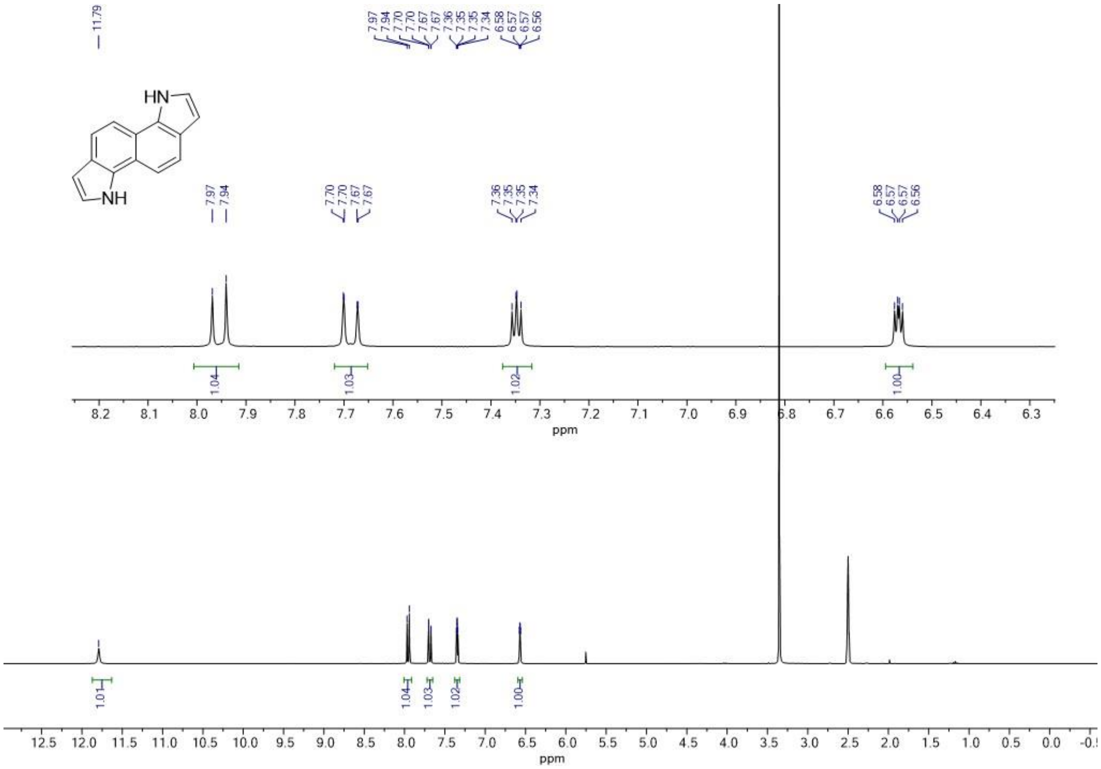

Figure S8: ^{1}H-NMR of **BI[6,7-*g*]**

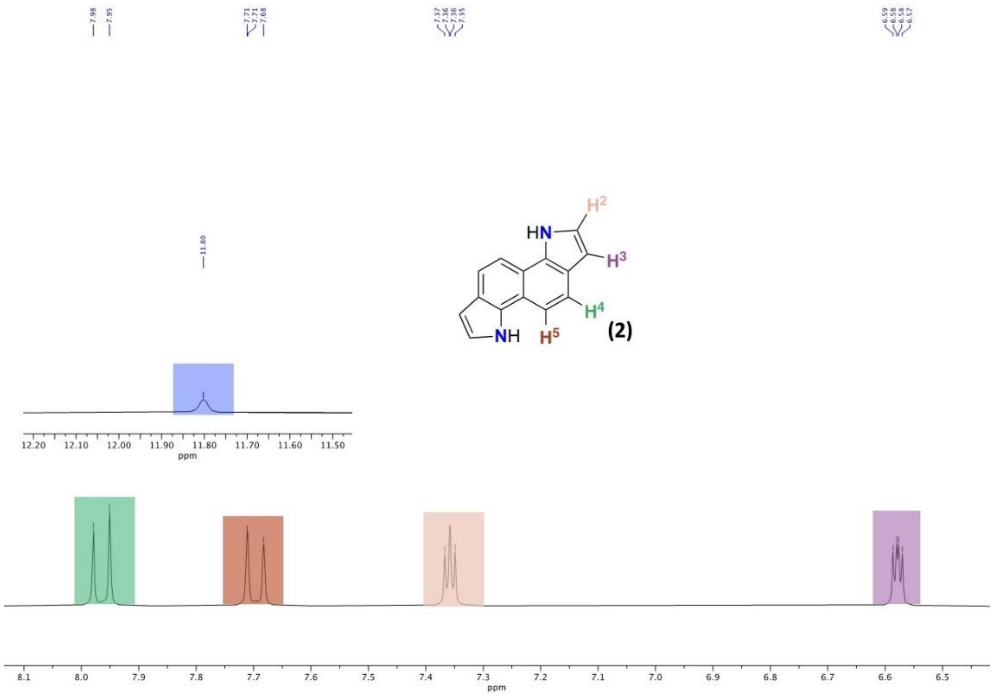

Figure S9: ^{1}H-NMR of **BI[6,7-*g*]**, aromatic domain, signal assignation.

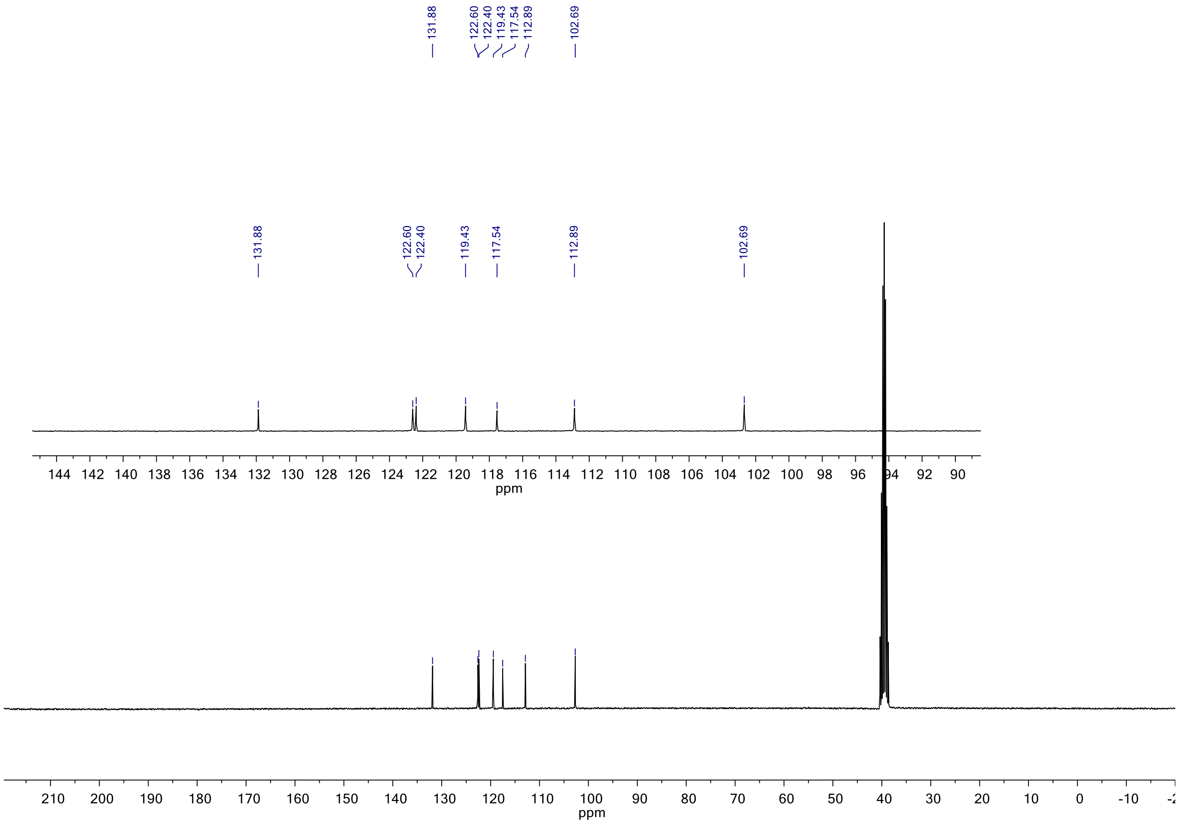

Figure S10: ^{13}C-NMR of **BI[6,7-*g*]**.

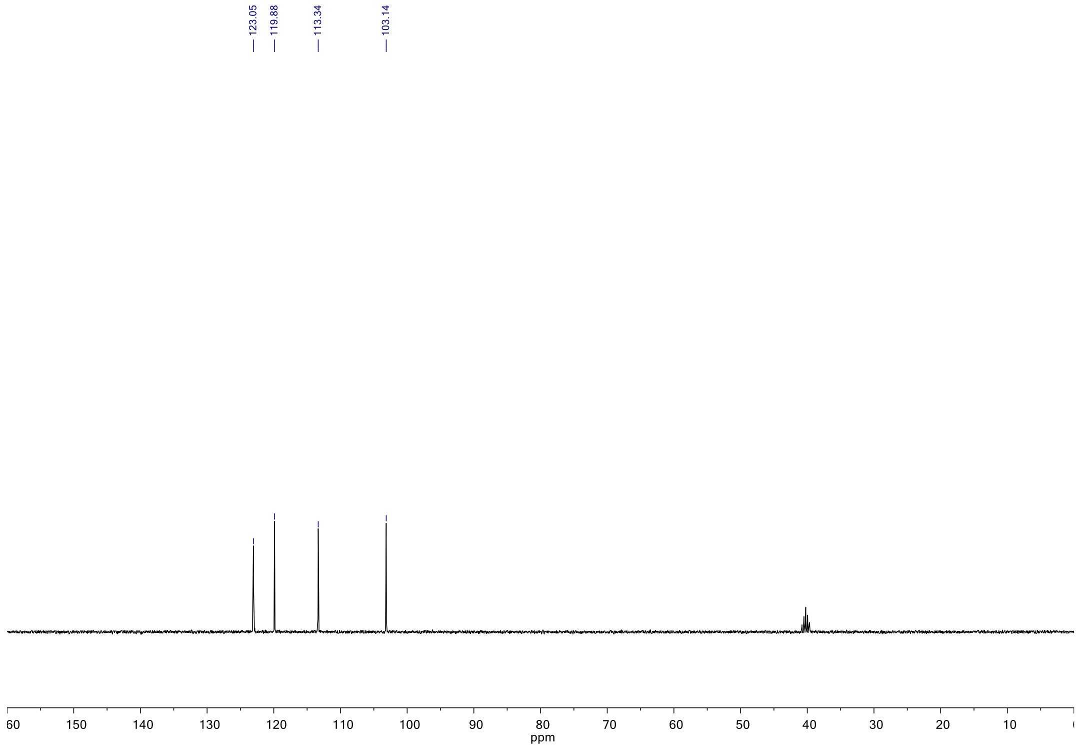

Figure S11: ^{13}C-DEPT135-NMR of **DAP16**.

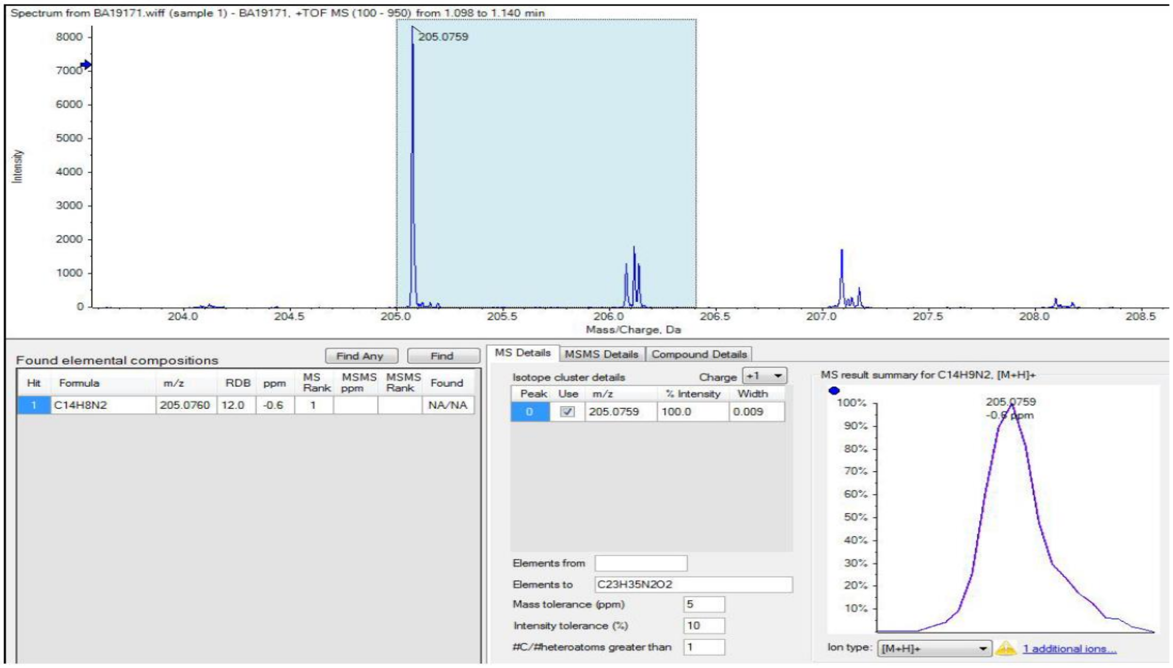

Figure S12: HRMS of **BI[6,7-*g*]**.

2. Symmetry Considerations for Pyrene (PG D_{2h})

Double (or higher order) excitations can be significant for optical transitions, enabled through simultaneous triplet excitations; a prominent example is the 2^1A_g state in oligoenes,[1,2] where double excitation is specifically accomplished by the very large exchange integral for $1B_u$ in polyenes. Therefore, oligoenes cannot be treated with standard TD-DFT methods. On the other hand, for other conjugated oligomers, this can be distinctively different, i.e. if the exchange integral is considerable smaller than in oligoenes.[3] For this reason, for instance, excited state absorption spectra of oligophenylenevinylenes are well calculated by TD-DFT.[3-5] The electronic situation of pyrene (**Py**) is distinctively different from oligoenes, so that low-lying symmetry-forbidden transitions do not play a role for the photophysics, see Tables S1, S2. On the other hand, alternant pairing of the (symmetry-allowed) $^1B_{2u}$ one-electron configurations $\Phi_{1,2}$ is essential; here, however, the splitting of the $1,2^1B_{2u}$ depends very sensitively on the method used. In particular, the good performance of multi-configurational methods[6,7] points to important double excitation contribution. In fact, $^1B_{2u}$ symmetry can be achieved by simultaneous excitation of 1^3B_{1u} and 1^3B_{3g}, see Figure S13. The necessary condition for a large exchange integral is fulfilled for $1B_{1u}$ (Tables S1, S2) resulting in $\Delta E(1^1B_{1u}$ $-1^3B_{1u}) = 1.54$ eV. Although the exchange integral for B_{3g} this might be sufficient to sensitively influence the spectral position of 1^1B_{2u}, and explains the rather unsatisfying performance of standard TD-DFT methods.

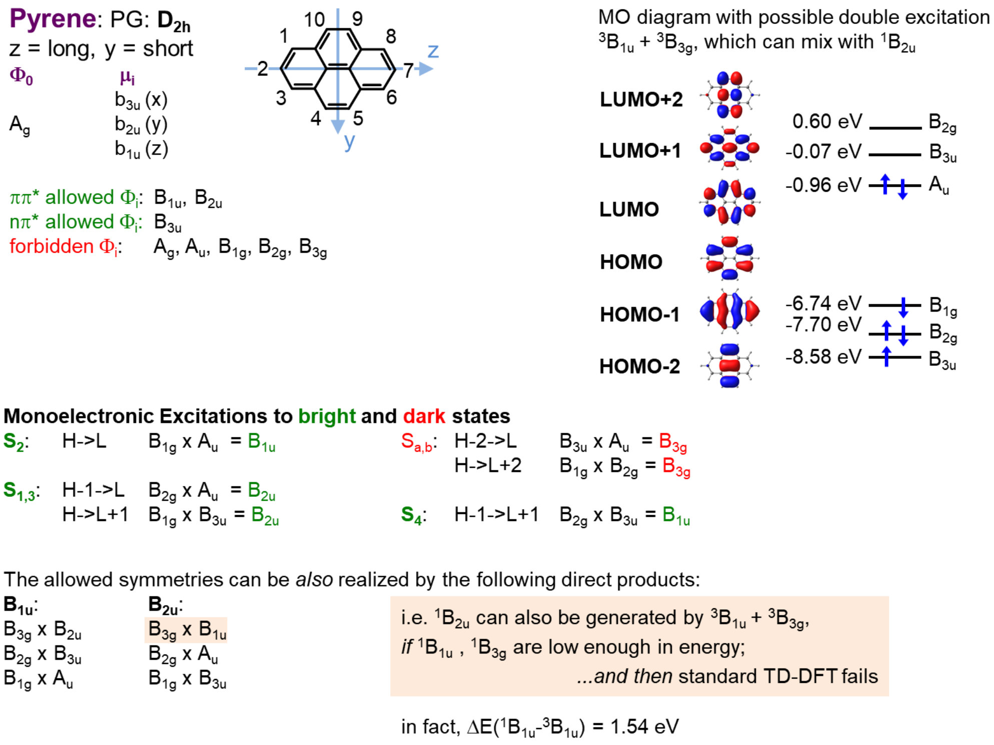

Figure S13: Symmetry considerations for Pyrene.

3. Additional Calculation Results

3.1. Vertical Transitions for DAP16, DAP27, Py

Table S1: Relevant electronic singlet transitions from S_0 (1^1A_g), giving symmetries, vertical energies E_{vert}, oscillator strengths *f*, and MO descriptions for the compounds under study, calculated at the TD-DFT level (M06-2X/6-311G(d,p)//M06-2X/6-311G(d,p)).

	Transition	Symmetry	E_{vert} (eV)	*f*	MO description	Contribution (%)
**DAP16**	S_1	1^1B_u	4.05	0.1739	H >L	59.17
					H-1 > L	23.51
					H > L+1	9.23
	S_2	1^1A_u	4.19	0.0024	H-2 > L	88.79
	S_3	2^1B_u	4.40	0.1257	H-1 > L	43.82
					H > L	31.07
					H > L+1	21.89
	S_4	1^1B_g	4.60	0.0000	H-4 > L	79.59
					H-2 > L+4	14.03
	S_5	2^1A_g	4.87	0.0000	H-3 > L	95.93
**DAP27**	S_1	1^1B_{2u}	4.02	0.0698	H > L+1	85.37
					H-1 > L	13.40
	S_2	1^1B_{1u}	4.17	0.3672	H > L	92.78
	S_3	1^1A_u	4.69	0.0000	H-2 > L	88.90
	S_4	2^1B_{2u}	4.74	0.0047	H-4 > L+4	13.02
					H-2 > L+1	82.86
	S_5	1^1B_{2g}	4.86	0.0000	H-4 > L+1	79.17
					H-2 > L+4	15.07
**Py**	S_1	1^1B_{2u}	4.07	0.0005	H-1 > L	44.43
					H > L+1	53.83
	S_2	1^1B_{1u}	4.12	0.3337	H > L	91.20
	S_3	1^1B_{3g}	4.87	0.0000	H > L+2	94.31
	S_4	2^1B_{2u}	5.11	0.3938	H-1 > L	53.65
					H > L+1	44.58
	S_5	2^1B_{3g}	5.20	0.0000	H-2 > L	91.31

Table S2: Relevant electronic triplet transitions from S_0 (1^1A_g), giving symmetries, vertical energies E_{vert} and MO descriptions for the compounds under study, calculated at the TD-DFT level (M06-2X/6-311G(d,p)//M06-2X/6-311G(d,p)).

Compound	Transition	Symmetry	E_{vert} (eV)	MO description	Contribution (%)
**DAP16**	T_1	1^3B_u	2.71	H >L	87.22
	T_2	2^3B_u	3.65	H-1 > L	88.46
	T_3	1^3A_u	3.78	H-2 > L	80.53
				H-4 > L+3	10.10
	T_4	1^3A_g	3.94	H-3 > L	67.22
				H > L+2	20.84
	T_5	1^3B_g	4.09	H-4 > L	70.47
				H-2 > L+3	16.03
**DAP27**	T_1	1^3B_{1u}	2.59	H > L	92.70
	T_2	1^3B_{2u}	3.42	H > L+1	97.33
	T_3	1^3B_{3g}	3.96	H-3 > L	35.43
				H > L+2	56.03
	T_4	1^3B_{3u}	4.19	H-4 > L+4	17.87
				H-2 > L+1	75.39
	T_5	1^3B_{2g}	4.27	H-4 > L+1	72.71
				H-2 > L+4	19.26
**Py**	T_1	1^3B_{1u}	2.58	H > L	92.25
	T_2	1^3B_{2u}	3.84	H-1 > L	13.36
				H > L+1	83.16
	T_3	2^3B_{2u}	3.91	H-1 > L	83.49
				H > L+1	13.96
	T_4	1^3B_{3g}	3.97	H-2 > L	36.48
				H > L+2	50.05
	T_5	1^3A_g	4.28	H-3 > L	42.57
				H > L+3	46.47

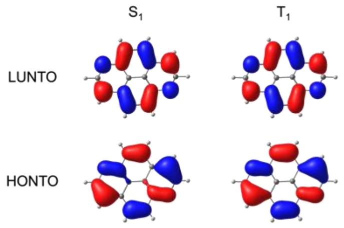

Figure S14: Natural transition orbitals of the first singlet and triplet state: S_1 (1^1B_u) and T_1 (1^3B_u) for **DAP16**, calculated at the TD-DFT level (M06-2X/6-311G(d,p)//M06-2X/6-311G(d,p)).

Table S3: Relevant electronic singlet and triplet transitions from S_0 (1^1A_g), giving symmetries (Symm.), vertical energies E_{vert}, oscillator strengths f, and MO descriptions for the compounds under study, calculated at the TD-DFT level (different functionals with the basis set 6-311G(d,p), PCM: DCM).

**DAP16: TD-DFT in DCM (PCM)**						**DAP27: TD-DFT in DCM (PCM)**						**Py: TD-DFT in DCM (PCM)**					
**Functional**	**Transition**	**Symm.**	**E_{vert} (eV)**	**E_{vert} (nm)**	***f***	**Functional**	**Transition**	**Symm.**	**E_{vert} (eV)**	**E_{vert} (nm)**	***f***	**Functional**	**Transition**	**Symm**	**E_{vert} (eV)**	**E_{vert} (nm)**	***f***
M06-2X	$S_0 \rightarrow S_1$	1^1B_u	3.98	311	0.3141	M06-2X	$S_0 \rightarrow S_1$	1^1B_{2u}	3.96	313	0.1157	M06-2X	$S_0 \rightarrow S_1$	1^1B_{1u}	4.04	307	0.4974
	$S_0 \rightarrow S_2$	1^1A_u	4.26	291	0.0028		$S_0 \rightarrow S_2$	1^1B_{1u}	4.07	304	0.5290		$S_0 \rightarrow S_2$	1^1B_{2u}	4.06	306	0.0020
	$S_0 \rightarrow S_3$	2^1B_u	4.37	284	0.1456		$S_0 \rightarrow S_3$	1^1B_{3u}	4.79	259	0.0053		$S_0 \rightarrow S_3$	1^1B_{3g}	4.84	256	0.0000
	$S_0 \rightarrow S_4$	1^1B_g	4.68	265	0.0000		$S_0 \rightarrow S_4$	1^1A_u	4.80	258	0.0000		$S_0 \rightarrow S_4$	2^1B_{2u}	5.01	248	0.5477
	$S_0 \rightarrow S_5$	1^1A_g	4.84	256	0.0000		$S_0 \rightarrow S_5$	1^1B_{3g}	4.89	253	0.0000		$S_0 \rightarrow S_5$	2^1B_{3g}	5.23	237	0.0000
wB97XD	$S_0 \rightarrow S_1$	1^1B_u	3.94	314	0.3112	wB97XD	$S_0 \rightarrow S_1$	1^1B_{2u}	3.93	316	0.1124	wB97XD	$S_0 \rightarrow S_1$	1^1B_{1u}	4.00	310	0.4843
	$S_0 \rightarrow S_2$	2^1B_u	4.32	287	0.1438		$S_0 \rightarrow S_2$	1^1B_{1u}	4.04	307	0.5194		$S_0 \rightarrow S_2$	1^1B_{2u}	4.03	308	0.0010
	$S_0 \rightarrow S_3$	1^1A_u	4.38	283	0.0028		$S_0 \rightarrow S_3$	1^1A_u	4.81	258	0.0000		$S_0 \rightarrow S_3$	1^1B_{3g}	4.82	257	0.0000
	$S_0 \rightarrow S_4$	1^1A_g	4.76	261	0.0000		$S_0 \rightarrow S_4$	1^1B_{3g}	4.87	255	0.0000		$S_0 \rightarrow S_4$	2^1B_{2u}	5.04	246	0.5802
	$S_0 \rightarrow S_5$	1^1B_g	4.80	258	0.0000		$S_0 \rightarrow S_5$	1^1B_{1g}	4.99	249	0.0000		$S_0 \rightarrow S_5$	2^1B_{3g}	5.16	240	0.0000
CAM-B3LYP	$S_0 \rightarrow S_1$	1^1B_u	3.93	315	0.3102	CAM-B3LYP	$S_0 \rightarrow S_1$	1^1B_{2u}	3.92	316	0.1114	CAM-B3LYP	$S_0 \rightarrow S_1$	1^1B_{1u}	3.98	311	0.4748
	$S_0 \rightarrow S_2$	2^1B_u	4.31	288	0.1354		$S_0 \rightarrow S_2$	1^1B_{1u}	4.02	308	0.5126		$S_0 \rightarrow S_2$	1^1B_{2u}	4.02	308	0.0011
	$S_0 \rightarrow S_3$	1^1A_u	4.41	281	0.0030		$S_0 \rightarrow S_3$	1^1A_u	4.82	257	0.0000		$S_0 \rightarrow S_3$	1^1B_{3g}	4.82	257	0.0000
	$S_0 \rightarrow S_4$	1^1A_g	4.77	260	0.0000		$S_0 \rightarrow S_4$	1^1B_{3g}	4.87	254	0.0000		$S_0 \rightarrow S_4$	2^1B_{2u}	5.02	247	0.5655
	$S_0 \rightarrow S_5$	1^1B_g	4.82	257	0.0000		$S_0 \rightarrow S_5$	1^1B_{1g}	5.00	248	0.0000		$S_0 \rightarrow S_5$	2^1B_{3g}	5.17	240	0.0000
D3-B3LYP	$S_0 \rightarrow S_1$	1^1B_u	3.68	337	0.2935	D3-B3LYP	$S_0 \rightarrow S_1$	1^1B_{2u}	3.69	336	0.0952	D3-B3LYP	$S_0 \rightarrow S_1$	1^1B_{1u}	3.68	337	0.3826
	$S_0 \rightarrow S_2$	1^1A_u	3.83	323	0.0018		$S_0 \rightarrow S_2$	1^1B_{1u}	3.71	334	0.4034		$S_0 \rightarrow S_2$	1^1B_{2u}	3.80	326	0.0011
	$S_0 \rightarrow S_3$	2^1B_u	4.02	309	0.0678		$S_0 \rightarrow S_3$	1^1A_u	4.17	297	0.0000		$S_0 \rightarrow S_3$	1^1B_{3g}	4.40	282	0.0000
	$S_0 \rightarrow S_4$	1^1B_g	4.26	291	0.0000		$S_0 \rightarrow S_4$	1^1B_{1g}	4.34	286	0.0000		$S_0 \rightarrow S_4$	2^1B_{2u}	4.57	271	0.3753
	$S_0 \rightarrow S_5$	1^1A_g	4.33	286	0.0000		$S_0 \rightarrow S_5$	1^1B_{3g}	4.47	277	0.0000		$S_0 \rightarrow S_5$	2^1B_{3g}	4.70	264	0.0000
PBE0	$S_0 \rightarrow S_1$	1^1B_u	3.76	330	0.3092	PBE0	$S_0 \rightarrow S_1$	1^1B_{2u}	3.76	330	0.1032	PBE0	$S_0 \rightarrow S_1$	1^1B_{1u}	3.77	329	0.4076
	$S_0 \rightarrow S_2$	1^1A_u	3.93	315	0.0019		$S_0 \rightarrow S_2$	1^1B_{1u}	3.80	326	0.4313		$S_0 \rightarrow S_2$	1^1B_{2u}	3.88	319	0.0015
	$S_0 \rightarrow S_3$	2^1B_u	4.11	302	0.0764		$S_0 \rightarrow S_3$	1^1A_u	4.31	288	0.0000		$S_0 \rightarrow S_3$	1^1B_{3g}	4.52	274	0.0000
	$S_0 \rightarrow S_4$	1^1B_g	4.38	283	0.0000		$S_0 \rightarrow S_4$	1^1B_{1g}	4.49	276	0.0000		$S_0 \rightarrow S_4$	2^1B_{2u}	4.69	265	0.4079
	$S_0 \rightarrow S_5$	1^1A_g	4.46	278	0.0000		$S_0 \rightarrow S_5$	1^1B_{3g}	4.59	270	0.0000		$S_0 \rightarrow S_5$	2^1B_{3g}	4.83	257	0.0000

DAP16: TD-DFT in DCM (PCM)						DAP27: TD-DFT in DCM (PCM)						Py: TD-DFT in DCM (PCM)					
**Functional**	**Transition**	**Symm.**	**E_{vert} (eV)**	**E_{vert} (nm)**	***f***	**Functional**	**Transition**	**Symm.**	**E_{vert} (eV)**	**E_{vert} (nm)**	***f***	**Functional**	**Transition**	**Symm.**	**E_{vert} (eV)**	**E_{vert} (nm)**	***f***
M06-2X	$S_0 \rightarrow T_1$	1^3B_u	2.70	460	0.0000	M06-2X	$S_0 \rightarrow T_1$	1^3B_{1u}	2.59	479	0.0000	M06-2X	$S_0 \rightarrow T_1$	1^3B_{1u}	2.60	478	0.0000
	$S_0 \rightarrow T_2$	2^3B_u	3.65	340	0.0000		$S_0 \rightarrow T_2$	1^3B_{2u}	3.36	369	0.0000		$S_0 \rightarrow T_2$	1^3B_{2u}	3.81	325	0.0000
	$S_0 \rightarrow T_3$	1^3A_u	3.86	321	0.0000		$S_0 \rightarrow T_3$	1^3B_{3g}	3.96	313	0.0000		$S_0 \rightarrow T_3$	2^3B_{2u}	3.92	316	0.0000
	$S_0 \rightarrow T_4$	1^3A_g	3.93	315	0.0000		$S_0 \rightarrow T_4$	1^3B_{3u}	4.27	290	0.0000		$S_0 \rightarrow T_4$	1^3B_{3g}	3.98	312	0.0000
	$S_0 \rightarrow T_5$	3^3Bu	4.09	303	0.0000		$S_0 \rightarrow T_5$	1^3A_g	4.31	288	0.0000		$S_0 \rightarrow T_5$	2^3B1u	4.30	288	0.0000
wB97XD	$S_0 \rightarrow T_1$	1^3B_u	2.30	539	0.0000	wB97XD	$S_0 \rightarrow T_1$	1^3B_{1u}	2.15	576	0.0000	wB97XD	$S_0 \rightarrow S_1$	1^3B_{1u}	2.15	576	0.0000
	$S_0 \rightarrow T_2$	2^3B_u	3.50	354	0.0000		$S_0 \rightarrow T_2$	1^3B_{2u}	3.26	380	0.0000		$S_0 \rightarrow S_2$	1^3B_{3g}	3.68	337	0.0000
	$S_0 \rightarrow T_3$	1^3A_g	3.62	342	0.0000		$S_0 \rightarrow T_3$	1^3B_{3g}	3.66	338	0.0000		$S_0 \rightarrow S_3$	1^3B_{2u}	3.75	331	0.0000
	$S_0 \rightarrow T_4$	1^3A_u	3.90	318	0.0000		$S_0 \rightarrow T_4$	1^3A_g	3.96	313	0.0000		$S_0 \rightarrow S_4$	2^3B_{2u}	3.84	323	0.0000
	$S_0 \rightarrow T_5$	3^3Bu	3.96	313	0.0000		$S_0 \rightarrow T_5$	2^3B_{1u}	4.34	286	0.0000		$S_0 \rightarrow S_5$	1^3A_g	3.94	314	0.0000
CAM-B3LYP	$S_0 \rightarrow T_1$	1^3B_u	2.18	570	0.0000	CAM-B3LYP	$S_0 \rightarrow T_1$	1^3B_{1u}	2.03	611	0.0000	CAM-B3LYP	$S_0 \rightarrow S_1$	1^3B_{1u}	2.24	555	0.0000
	$S_0 \rightarrow T_2$	2^3B_u	3.47	357	0.0000		$S_0 \rightarrow T_2$	1^3B_{2u}	3.23	384	0.0000		$S_0 \rightarrow S_2$	1^3B_{2u}	3.49	355	0.0000
	$S_0 \rightarrow T_3$	1^3A_g	3.55	350	0.0000		$S_0 \rightarrow T_3$	1^3B_{3g}	3.59	346	0.0000		$S_0 \rightarrow S_3$	1^3B_{3g}	3.61	343	0.0000
	$S_0 \rightarrow T_4$	1^3A_u	3.89	319	0.0000		$S_0 \rightarrow T_4$	1^3Ag	3.86	321	0.0000		$S_0 \rightarrow S_4$	2^3B_{2u}	3.65	340	0.0000
	$S_0 \rightarrow T_5$	3^3Bu	3.90	318	0.0000		$S_0 \rightarrow T_5$	2^3B_{1u}	4.26	291	0.0000		$S_0 \rightarrow S_5$	2^3B_{1u}	3.93	316	0.0000
D3-B3LYP	$S_0 \rightarrow T_1$	1^3B_u	2.35	528	0.0000	D3-B3LYP	$S_0 \rightarrow T_1$	1^3B_{1u}	2.22	557	0.0000	D3-B3LYP	$S_0 \rightarrow S_1$	1^3B_{1u}	2.10	590	0.0000
	$S_0 \rightarrow T_2$	2^3B_u	3.36	369	0.0000		$S_0 \rightarrow T_2$	1^3B_{2u}	3.09	401	0.0000		$S_0 \rightarrow S_2$	1^3B_{2u}	3.53	351	0.0000
	$S_0 \rightarrow T_3$	1^3A_u	3.44	360	0.0000		$S_0 \rightarrow T_3$	1^3B_{3g}	3.61	344	0.0000		$S_0 \rightarrow S_3$	1^3B_{3g}	3.56	349	0.0000
	$S_0 \rightarrow T_4$	1^3A_g	3.54	350	0.0000		$S_0 \rightarrow T_4$	1^3A_g	3.94	315	0.0000		$S_0 \rightarrow S_4$	2^3B_{2u}	3.70	336	0.0000
	$S_0 \rightarrow T_5$	3^3Bu	3.74	332	0.0000		$S_0 \rightarrow T_5$	1^3B_{3u}	3.97	312	0.0000		$S_0 \rightarrow S_5$	1^3A_g	3.85	322	0.0000
PBE0	$S_0 \rightarrow T_1$	1^3B_u	2.23	556	0.0000	PBE0	$S_0 \rightarrow T_1$	1^3B_{1u}	2.09	593	0.0000	PBE0	$S_0 \rightarrow S_1$	1^3B_{1u}	2.09	593	0.0000
	$S_0 \rightarrow T_2$	2^3B_u	3.38	367	0.0000		$S_0 \rightarrow T_2$	1^3B_{2u}	3.10	400	0.0000		$S_0 \rightarrow S_2$	1^3B_{2u}	3.10	400	0.0000
	$S_0 \rightarrow T_3$	1^3A_g	3.48	356	0.0000		$S_0 \rightarrow T_3$	1^3B_{3g}	3.54	350	0.0000		$S_0 \rightarrow S_3$	1^3B_{3g}	3.54	350	0.0000
	$S_0 \rightarrow T_4$	1^3A_u	3.49	356	0.0000		$S_0 \rightarrow T_4$	1^3A_g	3.86	321	0.0000		$S_0 \rightarrow S_4$	1^3A_g	3.86	321	0.0000
	$S_0 \rightarrow T_5$	3^3Bu	3.75	331	0.0000		$S_0 \rightarrow T_5$	1^3B_{3u}	4.00	310	0.0000		$S_0 \rightarrow S_5$	1^3B_{3u}	4.00	310	0.0000

3.2. Adiabatic Transitions for DAP16

Table S4: Relevant electronic transitions for **DAP16** in vacuum and DCM (PCM) at the TD-DFT level (M06-2X/6-311G(d,p)//M06-2X/6-311G(d,p)).

**Vac**	**E_{vert} (eV)**	**E_{adia} (eV)**	**E_{re} (eV)**
S_1 (1^1B_u)	4.05	3.90	0.15
S_2 (1^1A_u)	4.19	3.85	0.34
T_1 (1^3B_u)	2.71	2.25	0.46
T_2 (2^3B_u)	3.65	3.44	0.21
T_3 (1^3A_u)	3.78	3.48	0.30

**DCM**	**E_{vert} (eV)**	**E_{adia} (eV)**	**E_{re} (eV)**
S_1 (1^1B_u)	3.98	3.68	0.30
S_2 (1^1A_u)	4.26	3.93	0.33
T_1 (1^3B_u)	2.70	2.24	0.46
T_2 (2^3B_u)	3.65	3.43	0.22
T_3 (1^3A_u)	3.86	3.58	0.28

In vacuum, the stabilization of S_1 upon geometry optimization is underestimated at this level of theory, giving rise to a switch in adiabatic energies between S_1 and S_2, however, this inconsistency is corrected in the PCM (DCM) model, which is in reasonable agreement with the experimental observations.

3.3. SOC matrix elements and ISC Rates for DAP16, DAP27, Py

The transition between states with different multiplicities become possible through spin-orbit coupling (SOC). In the case of inter-system crossing (ISC) in the frame of Fermi's golden rule, the SOC operator is described as

$$k_{ISC} = \frac{2\pi}{\hbar} {V_{SOC}}^2 \rho_E \quad (1)$$

where ρ_E can be described as the Frank-Condon weighted density of states, V_{SOC} is the SOC matrix elements. The individual k_{ISC} is given by,

$$k_{ISC;m,n} = \frac{2\pi}{\hbar} \frac{{V_{SOC}}^2(m,n)}{\sqrt{4\pi\lambda_{n(m)}k_B T}} e^{-\frac{(\lambda_{n(m)}-\Delta E_{m,n})^2}{4\pi\lambda_{n(m)}k_B T}} \quad (2)$$

where $\Delta E_{m,n}$ is the adiabatic energies between the singlet m and triplet n states, k_B is the Boltzmann constant and T is the temperature. Specifically, $\lambda_{n(m)}$ is energy variation in the initial state that is, switching from the singlet equilibrium geometry to the triplet equilibrium geometry.

Table S5a: Calculated ISC rates k_{ISC} from the initial S_i state to the final T_f state for **DAP16** at the TD-DFT level (M06-2X/6-311G(d,p)//M06-2X/6-311G(d,p)).

Singlets	Triplets	ΔE(S_i-T_f) (eV)	V_{SOC} (eV)[)]	k_{ISC} (s^{-1})
S_1 (1^1B_u)	1^3B_u	-1.64	$4.96\cdot10^{-6}$	0 [a)]
	1^3A_u	-0.41	$3.36\cdot10^{-4}$	$1.84\cdot10^{10}$
	2^3B_u	-0.46	$2.48\cdot10^{-6}$	$1.83\cdot10^{4}$
S_2 (1^1A_u)	1^3B_u	-1.60	$1.16\cdot10^{-3}$	$3.76\cdot10^{1}$
	1^3A_u	-0.37	$1.36\cdot10^{-5}$	0 [a)]
	2^3B_u	-0.41	$7.79\cdot10^{-4}$	$9.49\cdot10^{10}$

[a)] calculated k_{ISC} values are negligible.

Table S5b: Calculated ISC rates k_{ISC} from the initial S_i state to the final T_f state for **DAP27** at the TD-DFT level (M06-2X/6-311G(d,p)//M06-2X/6-311G(d,p)).

Singlets	Triplets	ΔE(S_i-T_f) / eV	V_{SOC} (eV)	k_{ISC} (s^{-1})
S_1 (1^1B_{2u})	1^3B_{1u}	-1.73	$1.05\cdot10^{-5}$	0 [a)]
	1^3B_{2u}	-0.61	0 [b)]	—
	1^3B_{3g}	-0.19	0 [b)]	—
	1^3B_{3u}	0.17	$1.03\cdot10^{-4}$	$1.66\cdot10^{6}$
	1^3B_{2g}	0.21	0 [b)]	—
S_2 (1^1B_{1u})	1^3B_{1u}	-1.80	0 [b)]	—
	1^3B_{2u}	-0.69	$1.05\cdot10^{-5}$	$5.00\cdot10^{3}$
	1^3B_{3g}	-0.27	0 [b)]	—
	1^3B_{3u}	0.09	$3.61\cdot10^{-4}$	$3.19\cdot10^{7}$
	1^3B_{2g}	0.13	0 [b)]	—

[a)] calculated k_{nr} values negligible. [b)] calculated V_{SOC} values negligible; below default threshold.

Table S5c: Calculated ISC rates k_{ISC} from the initial S_i state to the final T_f state for **Py** at the TD-DFT level (M06-2X/6-311G(d,p)//M06-2X/6-311G(d,p)).

Singlets	Triples	ΔE(S_i-T_f) (eV)	V_{SOC} (eV)	k_{ISC} (s^{-1})
S_1 (1^1B_{2u})	1^3B_{1u}	-1.77	$5.26\cdot10^{-6}$	0 [a)]
	1^3B_{2u}	-0.25	0 [b)]	—
	2^3B_{2u}	-0.18	0 [b)]	—
	1^3B_{3g}	-0.23	0 [b)]	—
	2^3B_{1u}	0.03	$3.51\cdot10^{-6}$	$1.36\cdot10^{5}$
	1^3A_g	0.20	0 [b)]	—
S_2 (1^1B_{1u})	1^3B_{1u}	-1.74	0 [b)]	
	1^3B_{2u}	-0.23	$7.01\cdot10^{-6}$	$1.08\cdot10^{7}$
	2^3B_{2u}	-0.15	$1.75\cdot10^{-6}$	$7.68\cdot10^{5}$
	1^3B_{3g}	-0.20	0 [b)]	—
	2^3B_{1u}	0.06	0 [b)]	—
	1^3A_g	0.23	0 [b)]	—

[a)] calculated k_{nr} values negligible. [b)] calculated V_{SOC} values negligible; below default threshold.

3.4. MECP Calculations for DAP16

The MECPs (minimal energy crossing points) were located by the program developed by Harvey and coworkers,[8] and modified by Tian Lu.[9] The structures were found by M06-2X//6311G(d,p) in vacuum. Two MECPs were found, geometrical distortions occur either in the N-containing ring (MECP 1), or in the N-free ring (MECP 2), respectively. In both cases the steric hinderance is rather low, and the energy difference between the relaxed excited states and the MECPs is only -0.02, and 0.03 eV; therefore, the transitions can be considered as barrierless. In Scheme 2, MECP 1 was adopted due to the lower energy. Table S5 shows the optimized geometry details at ground, excited states and the singlet-triplet MECP. For the above-mentioned optimized states, the structures maintain the symmetry of C_{2h}. At the singlet-triplet MECP, a slight distortion occurs in one N-containing ring, consequently breaking the symmetry. Geometry in the Cartesian system is given in Table S6.

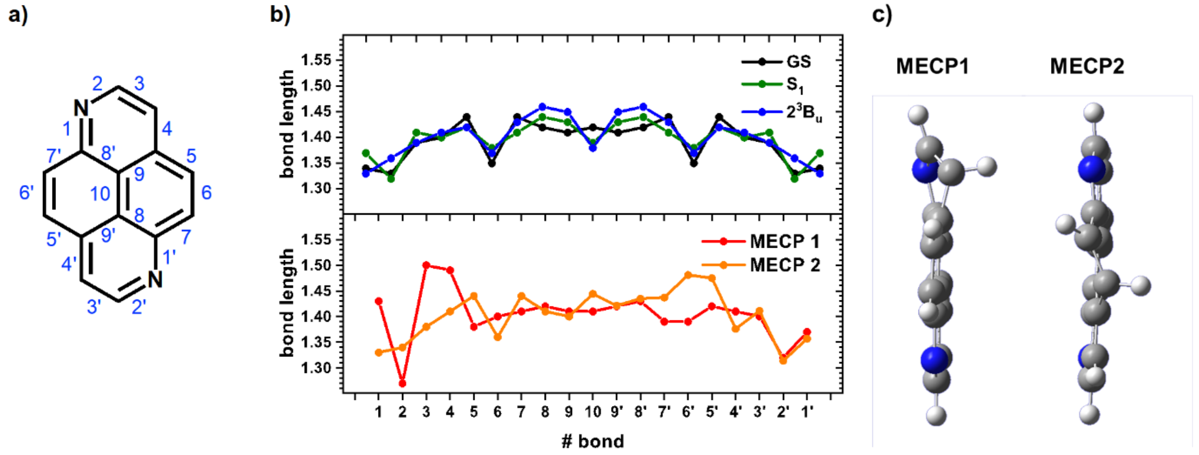

Figure S15: Geometries of **DAP16**, calculated for the ground state (GS; S_0), S_1, and T_2 (2^3B_u), and at the MECPs.

Table S6: Descriptions of the bond length variations in vacuum (unit: Å).

	1	2	3	4	5	6	7	8	9	10	9'	8'	7'	6'	5'	4'	3'	2'	1'
**GS**	1.34	1.33	1.39	1.40	1.44	1.35	1.44	1.42	1.41	1.42	1.41	1.42	1.44	1.35	1.44	1.40	1.39	1.33	1.34
**S_1**	1.37	1.32	1.41	1.40	1.42	1.38	1.41	1.44	1.43	1.39	1.43	1.44	1.41	1.38	1.42	1.40	1.41	1.32	1.37
**1^3B_u**	1.40	1.32	1.40	1.44	1.39	1.42	1.39	1.43	1.42	1.41	1.42	1.43	1.39	1.42	1.39	1.44	1.4	1.32	1.40
**1^3A_u**	1.34	1.34	1.37	1.42	1.42	1.37	1.42	1.44	1.42	1.42	1.42	1.44	1.42	1.37	1.42	1.42	1.37	1.34	1.34
**2^3B_u**	1.33	1.36	1.39	1.41	1.42	1.37	1.43	1.46	1.45	1.38	1.45	1.46	1.43	1.37	1.42	1.41	1.39	1.36	1.33
**MECP 1**	1.43	1.27	1.50	1.49	1.38	1.40	1.41	1.42	1.41	1.41	1.42	1.43	1.39	1.39	1.42	1.41	1.40	1.32	1.37
**MECP 2**	1.33	1.34	1.38	1.41	1.44	1.36	1.44	1.41	1.40	1.44	1.42	1.44	1.44	1.48	1.48	1.38	1.41	1.31	1.36

Table S7. Geometry of the singlet-triplet MECPs in the Cartesian coordinate system (unit: Å).

	MECP1			MECP2		
	X	Y	Z	X	Y	Z
C	2.633	-2.049	0.763	2.668	-1.992	0.910
H	3.460	-2.712	1.001	3.478	-2.642	1.233
C	2.910	-0.746	0.328	2.974	-0.758	0.297
C	1.843	0.114	0.021	1.939	0.059	-0.094
C	0.361	-1.757	0.613	0.419	-1.649	0.722
C	0.534	-0.411	0.193	0.610	-0.384	0.142
C	1.979	1.433	-0.481	2.035	1.284	-0.910
C	-0.593	0.394	-0.061	-0.532	0.413	-0.239
C	0.862	2.176	-0.848	0.959	2.282	-0.708
C	-0.425	1.689	-0.650	-0.384	1.771	-0.681
H	3.937	-0.417	0.216	4.005	-0.474	0.129
H	2.974	1.839	-0.627	2.297	1.084	-1.951
C	-2.059	-1.450	0.483	-1.992	-1.403	0.518
H	0.972	3.140	-1.329	1.152	3.302	-0.401
H	-3.058	-1.848	0.616	-3.000	-1.780	0.658
C	-0.946	-2.266	0.716	-0.921	-2.142	0.894
H	-1.062	-3.301	1.013	-1.029	-3.125	1.338
C	-1.887	-0.121	0.148	-1.826	-0.089	-0.042
C	-2.688	1.978	-0.966	-2.654	2.051	-0.762
C	-2.908	0.970	0.119	-2.910	0.758	-0.357
H	-3.530	2.431	-1.486	-3.475	2.727	-0.981
H	-3.025	1.462	1.089	-3.929	0.407	-0.250
N	-1.516	2.390	-1.240	1.450	-2.440	1.112
N	1.425	-2.565	0.900	-1.424	2.574	-0.901

Table S8: Descriptions of the bond length variations in vacuum. Relevant electronic transitions for **DAP16** in vacuum and DCM (PCM) at the TD-DFT level (M06-2X/6-311G(d,p)//M06-2X/6-311G(d,p)).

Electronic states	Electronic energy (Hartree)	ΔE_{EI} (eV)	$\Delta E_{MECP\text{-}GS}$ $\Delta E_{MECP\text{-}opted\ ES}$ (eV)
MECP 1_GS	-647.61		3.42
MECP 1 _Tn	-647.61	0.00	-0.02
MECP 2_GS	-647.61		3.47
MECP 2_Tn	-647.61	0.00	0.03

4. Additional Spectroscopic Results

4.1. Fluorescence and Phosphorescence Time Traces

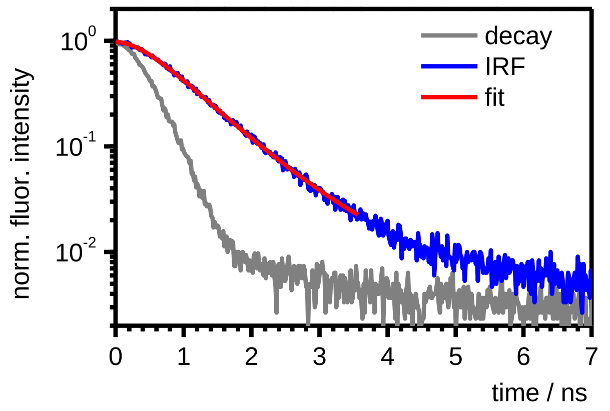

Figure S16: Fluorescence decay (blue) of **DAP16** in DCM at RT, λ_{ex} = 375 nm, λ_{det} = 374 nm. The mono-exponential fit (τ = 0.73 ns, red) was obtained by deconvolution with the instrumental response function (IRF, grey).

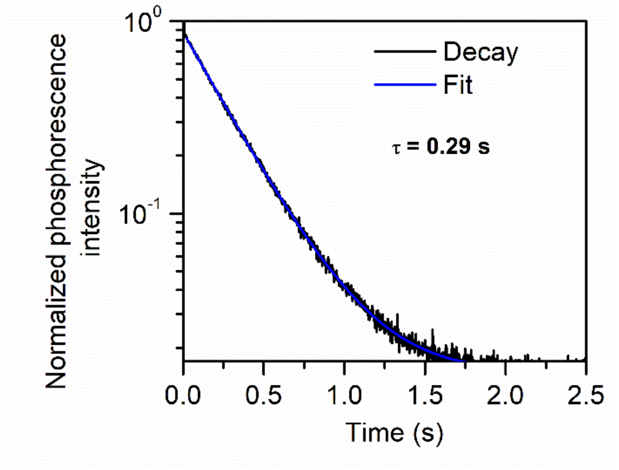

Figure S17: Phosphorescence decay (black) of DAP16 in 1wt% PMMA doped film at LT (65 K), λ_{ex} = 375 nm, λ_{det} = 564 nm nm. The mono-exponential fit (τ = 0.29 s, blue) was obtained by tail fitting.

4.2. Low temperature measurements of DAP16 and DAP27 doped in PMMA

We prepared 1wt% polymethyl methacrylate (PMMA) doped film of the two substrates (**DAP16** and **DAP27**), and measured the photophysical properties. For phosphorescence measurement, samples were mounted in a Magnex Scientific needle valve optical cryostat operated with liquid nitrogen. The nitrogen was pumped down to 65 K and allowed to warm up slowly to remove bubbles. The emission of the sample was dispersed in wavelength and detected either by a Princeton Instruments Spec10:400BR CCD camera or by a low dark current hybrid photomultiplier (PMA 06, PicoQuant), both attached to the Acton SP2500 spectrometer. A 355 nm Nd:YAG laser with a pulse width of 300 ps (Teemphotonics) was used as an excitation source. A 375 nm laser (Picoquant) was used as an excitation source and was operated in pulsed mode for fluorescence decay measurements and in continuous mode for the acquisition of ungated as well as gated PL spectra and for phosphorescence decay measurements. Fluorescence decay histograms were obtained using a HydraHarp TCSPC electronics (Picoquant), whereas phosphorescence decays were acquired using a TimeHarp multichannel scaler (Picoquant). In order to acquire gated spectra and to measure the phosphorescence decay a mechanical shutter (rise and decay times < 10ms, Thorlabs) was used to gate the laser. Trigger pulses for the shutter, the CCD camera and the TimeHarp electronics were provided by a Stanford Research Systems DG645 pulse and delay generator with 5 ps resolution.

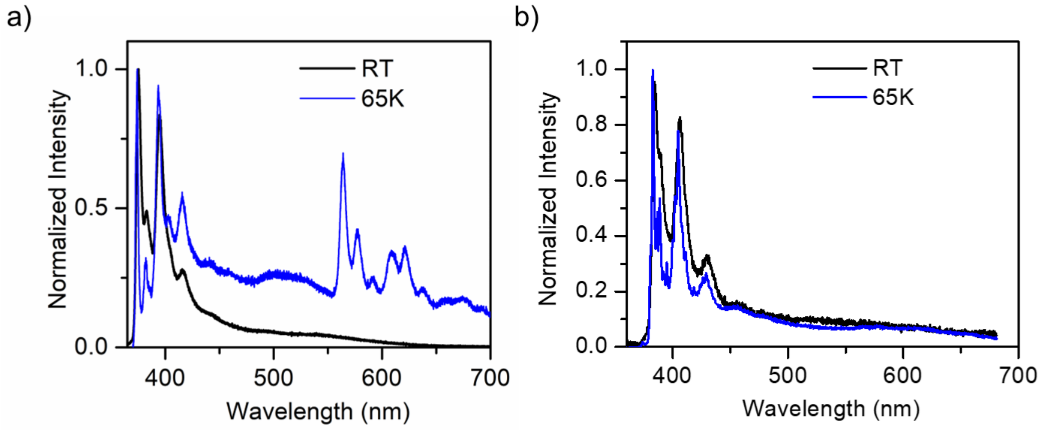

Figure S18: Normalized emission of 1wt% doped (in PMMA) at RT and 65 K of (a) **DAP16** and (b) **DAP27**

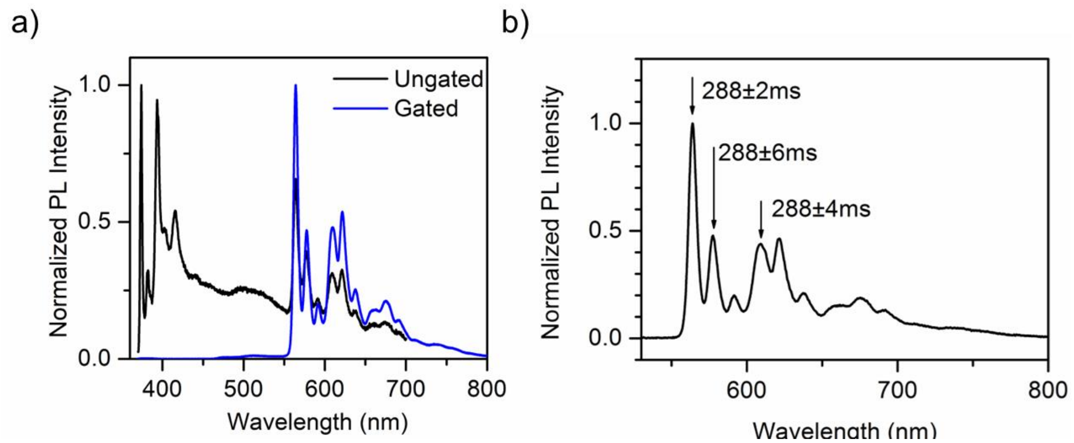

Figure S19: (a) Ungated and gated (phosphorescence) emission spectra of **DAP16**, and (b) phosphorescence lifetimes of **DAP16**.

5. Photochemistry of DAP16

5.1. Experimental

We prepared **DAP16** solution in DCM (and in $CHCl_3$) at a concentration of 5×10^{-6} M, and then irradiate the solution (unpurged and purged with Argon) using_355 nm Nd:YAG laser at optical density = 0, and measure the UV-Vis absorption and emission at a different time interval.

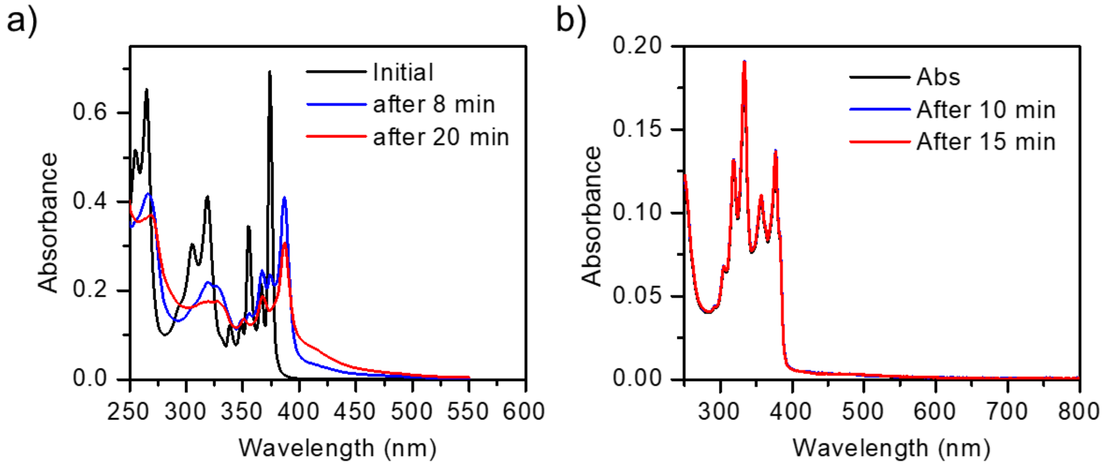

Figure S20: UV-vis absorption spectra of the **DAP16** (a) and **DAP27** (b) in DCM during 355 nm laser excitation.

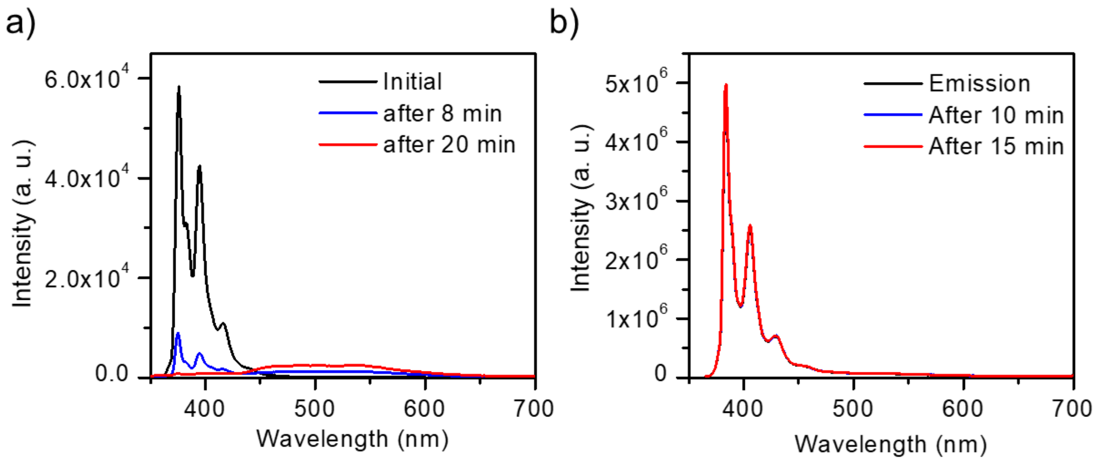

Figure S21: Emission spectra of the **DAP16** (a) and **DAP27**(b) in DCM during 355 nm laser excitation.

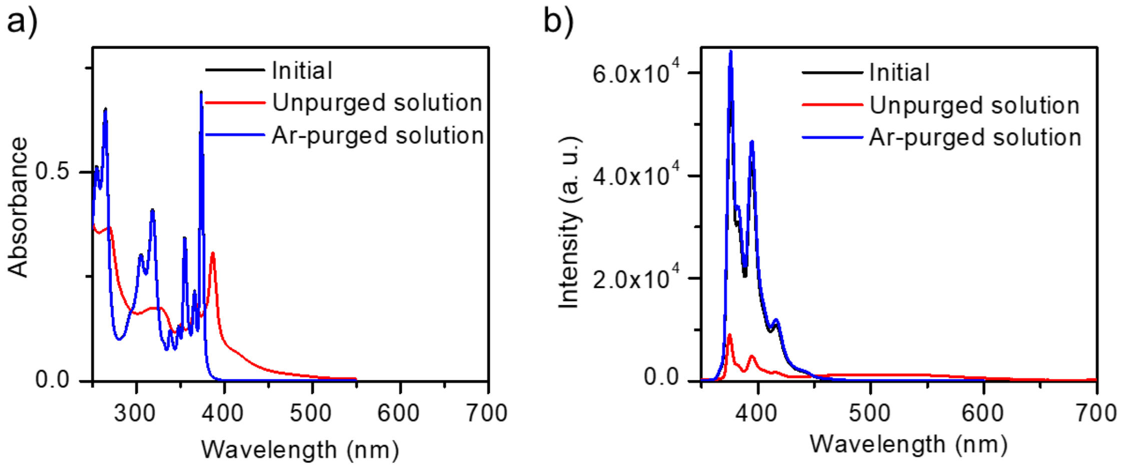

Figure S22: Comparison of UV-vis absorption spectra (a) and emission spectra (b) of **DAP16** in DCM under variable conditions after 355 nm laser irradiation.

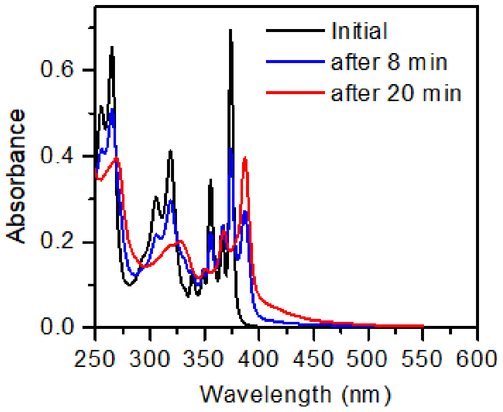

Figure S23: UV-vis absorption spectra of the **DAP16** in DCM (N_2-purged solution) during 355 nm laser excitation.

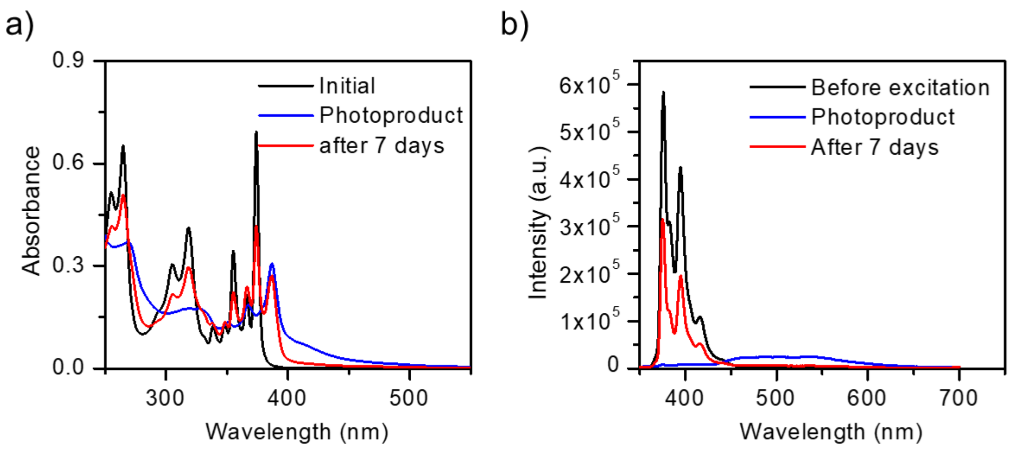

Figure S24: UV-vis absorption (a) and emission (b) spectra of **DAP16** in DCM after 7 days of the formed photoproduct.

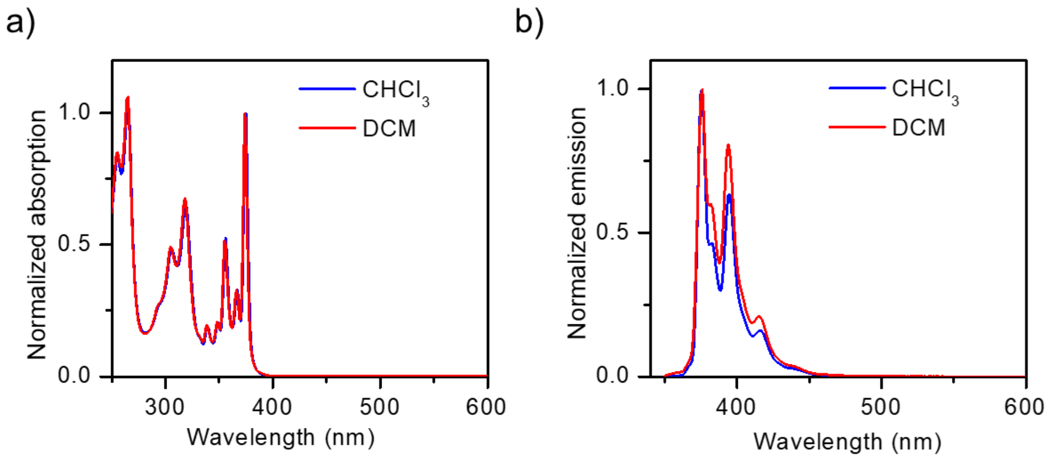

Figure S25: Normalized UV-vis absorption spectra (a) and emission spectra (b) of **DAP16** in different solvent.

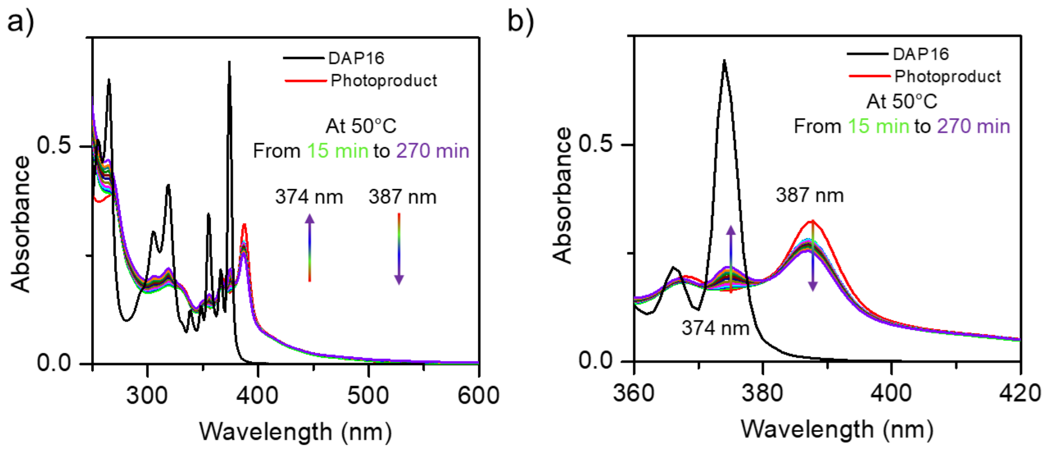

Figure S26: Thermally induced reversibility of the **DAP16** photoproduct. Full spectral range (from 250 nm to 600 nm) with variable time from 15 min to 270 min at 50°C (a), and enraged area (from 360 nm to 420 nm) (b).

4.2. Possible Photoproducts of DAP16

We calculated possible photoproducts of **DAP16** by DFT, including (i) noncovalent complex in different configurations (Table S8), (ii) various cycloadditions (Table S9), (iii) oxidation in different positions (Table S10), as well as (iv) peroxide formation (Table S11). The electronic ground state geometries of the possible photoproducts and their single point TD-DFT calculations were obtained by M06-2X//6311G(d,p) in DCM.

Reaction pathways (see Figure S22) can represent a potential energy surface represent reactants, intermediates, and products of a reaction. The position of the minimum in a valley represents the equilibrium structure. The energy difference between the product valley and reactant valley minima represents the energy of the reaction. The highest point on this lowest energy reaction path is the transition state (TS) for the reaction, and the difference between the energy of the TS and the reactant is the energy barrier for the reaction. All energy minima were confirmed by the absence of imaginary frequencies, whereas the transition states were characterized by one imaginary frequency with a propermode, referring to a saddle point along the potential energy surface and reflecting the dynamic pathway between two local maxima.

Table S9: Geometries of photoproducts concerning noncovalent complex in different configurations [left]; and relevant electronic singlet transitions from S_0, giving vertical energies E_{vert}, oscillator strengths *f* for the compounds under study at the TD-DFT level (M06-2X/6-311G(d,p)//M06-2X/6-311G(d,p) PCM: DCM) [right].

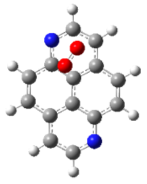

DAP16_top_asso

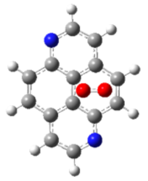

DAP16_edge_asso

DAP16_top_asso	E_{vert} (eV)	E_{vert} (nm)	*f*
S0->S1	0.52	2391	0.0001
S0->S2	3.17	391	0.0545
S0->S3	3.74	332	0.0534
S0->S4	4.05	306	0.2732
S0->S5	4.12	301	0.0066
S0->S6	4.29	289	0.0033
S0->S7	4.36	285	0.1030
S0->S8	4.44	279	0.0644
S0->S9	4.68	265	0.0001
S0->S10	4.83	257	0.0014

DAP16_edge_asso	E_{vert} (eV)	E_{vert} (nm)	*f*
S0->S1	0.47	2619	0.0001
S0->S2	3.00	413	0.0165
S0->S3	3.55	350	0.0036
S0->S4	3.98	311	0.3200
S0->S5	4.23	293	0.0011
S0->S6	4.29	289	0.0798
S0->S7	4.34	286	0.0005
S0->S8	4.37	284	0.1355
S0->S9	4.70	264	0.0001
S0->S10	4.90	253	0.0006

Table S10: Geometries of photoproducts concerning various cycloadditions [left]; and relevant electronic singlet transitions from S_0, giving vertical energies E_{vert}, oscillator strengths *f* for the compounds under study at the TD-DFT level (M06-2X/6-311G(d,p)//M06-2X/6-311G(d,p) PCM: DCM) [right].

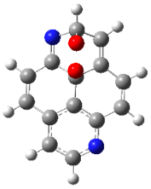

DAP16_top_cycla-in

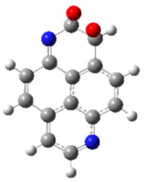

DAP16_top_cycla-out

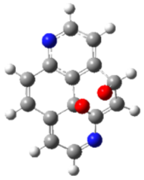

DAP16_edge_cycla1

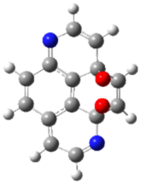

DAP16_edge_cycla2

DAP16_top_cycla-in	E_{vert} (eV)	E_{vert} (nm)	*f*
S0->S1	3.40	365	0.0006
S0->S2	3.58	346	0.0054
S0->S3	4.24	292	0.0255
S0->S4	4.37	283	0.3639
S0->S5	4.49	276	0.0184
S0->S6	4.70	264	0.0045
S0->S7	4.87	255	0.0091
S0->S8	5.00	248	0.0927
S0->S9	5.20	238	0.0988
S0->S10	5.35	232	0.2547

DAP16_top_cycla-out	E_{vert} (eV)	E_{vert} (nm)	*f*
S0->S1	3.41	364	0.1146
S0->S2	3.66	339	0.1101
S0->S3	4.14	299	0.0886
S0->S4	4.19	296	0.0028
S0->S5	4.40	281	0.0126
S0->S6	4.58	271	0.0171
S0->S7	4.67	266	0.0424
S0->S8	5.35	232	0.5716
S0->S9	5.57	223	0.0809
S0->S10	5.73	216	0.0054

DAP16_edge_cycla1	E_{vert} (eV)	E_{vert} (nm)	*f*
S0->S1	3.30	376	0.0811
S0->S2	3.52	352	0.0437
S0->S3	4.08	304	0.0770
S0->S4	4.30	288	0.0459
S0->S5	4.66	266	0.0282
S0->S6	4.82	257	0.2215
S0->S7	5.01	247	0.0413
S0->S8	5.12	242	0.0220
S0->S9	5.27	235	0.0453
S0->S10	5.57	223	0.1436

DAP16_edge_cycla1	E_{vert} (eV)	E_{vert} (nm)	*f*
S0->S1	2.71	458	0.2449
S0->S2	2.88	431	0.0301
S0->S3	3.47	357	0.0047
S0->S4	3.60	345	0.0181
S0->S5	3.80	327	0.0010
S0->S6	4.45	279	0.1336
S0->S7	4.61	269	0.0132
S0->S8	4.92	252	0.0609
S0->S9	5.21	238	0.0007
S0->S10	5.24	237	0.0932

Table S11: Geometries of photoproducts concerning oxidation in different positions [left]; and relevant electronic singlet transitions from S_0, giving vertical energies E_{vert}, oscillator strengths *f* for the compounds under study at the TD-DFT level (M06-2X/6-311G(d,p)//M06-2X/6-311G(d,p) PCM: DCM) [right].

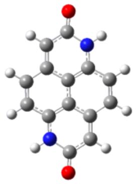

DAP16_ox1

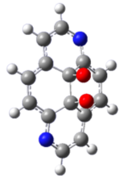

DAP16_ox2

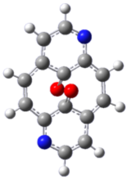

DAP16_ox3

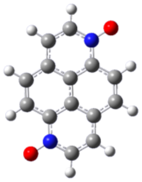

DAP16_ox4

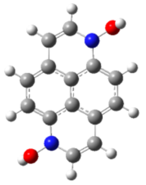

DAP16_ox5

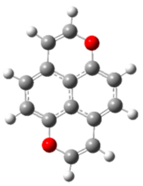

DAP16_ox6

DAP16_ox1	E_{vert} (eV)	E_{vert} (nm)	*f*
S0->S1	2.53	490	0.2739
S0->S2	4.19	296	0.0000
S0->S3	4.32	287	0.0000
S0->S4	4.50	276	0.0295
S0->S5	4.64	267	0.0000
S0->S6	4.66	266	0.0002
S0->S7	4.67	265	0.0000
S0->S8	4.75	261	0.0000
S0->S9	5.22	238	1.7380
S0->S10	5.92	210	0.0001

DAP16_ox2	E_{vert} (eV)	E_{vert} (nm)	*f*
S0->S1	2.47	502	0.0273
S0->S2	2.72	456	0.0834
S0->S3	3.16	392	0.0000
S0->S4	3.60	344	0.4904
S0->S5	3.77	328	0.0000
S0->S6	4.03	308	0.7699
S0->S7	4.22	294	0.0000
S0->S8	4.30	288	0.0395
S0->S9	4.35	285	0.0032
S0->S10	4.50	276	0.0740

DAP16_ox3	E_{vert} (eV)	E_{vert} (nm)	*f*
S0->S1	2.35	528	0.0874
S0->S2	2.83	439	0.0288
S0->S3	3.01	412	0.0003
S0->S4	3.39	366	0.0000
S0->S5	3.45	359	0.3170
S0->S6	3.94	315	0.6085
S0->S7	4.24	293	0.0017
S0->S8	4.33	286	0.0001
S0->S9	4.89	254	0.0433
S0->S10	4.91	253	0.0003

DAP16_ox4	E_{vert} (eV)	E_{vert} (nm)	*f*
S0->S1	3.20	388	0.6252
S0->S2	3.57	348	0.0407
S0->S3	4.09	303	0.0000
S0->S4	4.17	298	0.0000
S0->S5	4.20	295	0.0000
S0->S6	4.42	281	0.0000
S0->S7	4.67	265	0.4982
S0->S8	4.95	250	0.0000
S0->S9	5.04	246	0.0000
S0->S10	5.11	243	0.0000

DAP16_ox5	E_{vert} (eV)	E_{vert} (nm)	*f*
S0->S1	2.70	459	0.0000
S0->S2	3.07	403	0.0982
S0->S3	3.50	354	0.3788
S0->S4	4.90	253	0.2338
S0->S5	5.07	244	0.0000
S0->S6	5.23	237	0.0070
S0->S7	5.60	222	0.0000
S0->S8	5.64	220	0.0042
S0->S9	5.68	218	0.1246
S0->S10	5.76	215	0.0000

DAP16_ox6	E_{vert} (eV)	E_{vert} (nm)	*f*
S0->S1	3.51	353	0.4819
S0->S2	3.85	322	0.0100
S0->S3	4.14	299	0.0001
S0->S4	4.45	279	0.0016
S0->S5	4.66	266	0.4846
S0->S6	4.78	259	0.0013
S0->S7	4.88	254	0.0059
S0->S8	5.34	232	0.1634
S0->S9	5.47	227	0.0003
S0->S10	5.47	227	0.0404

Table S12: Geometries of photoproducts concerning peroxide formation [left]; and relevant electronic singlet transitions from S_0, giving vertical energies E_{vert}, oscillator strengths *f* for the compounds under study at the TD-DFT level (M06-2X/6-311G(d,p)//M06-2X/6-311G(d,p) PCM: DCM) [right].

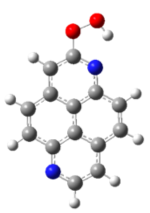

DAP16_top-in

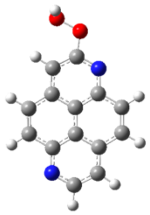

DAP16_top-out

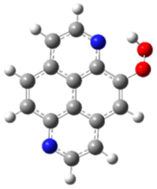

DAP16_edge-in

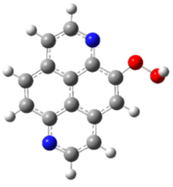

DAP16_edge-out

DAP16_top-in	E_{vert} (eV)	E_{vert} (nm)	*f*
S0->S1	3.71	334	0.3020
S0->S2	4.32	287	0.0018
S0->S3	4.35	285	0.1530
S0->S4	4.73	262	0.0018
S0->S5	5.19	239	0.5388
S0->S6	5.30	234	0.2338
S0->S7	5.32	233	0.0001
S0->S8	5.40	230	0.0000
S0->S9	5.60	222	0.2851
S0->S10	5.61	221	0.0007

DAP27_top-out	E_{vert} (eV)	E_{vert} (nm)	*f*
S0->S1	3.82	325	0.2722
S0->S2	4.33	287	0.0182
S0->S3	4.33	286	0.1986
S0->S4	4.77	260	0.0011
S0->S5	4.97	249	0.0002
S0->S6	5.25	236	0.4800
S0->S7	5.39	230	0.1168
S0->S8	5.59	222	0.0012
S0->S9	5.67	219	0.9713
S0->S10	5.74	216	0.1404

DAP16_edge-in	E_{vert} (eV)	E_{vert} (nm)	*f*
S0->S1	3.97	312	0.3277
S0->S2	4.24	292	0.1303
S0->S3	4.34	286	0.0130
S0->S4	4.62	268	0.0160
S0->S5	4.83	257	0.0005
S0->S6	5.14	241	0.4912
S0->S7	5.35	232	0.0094
S0->S8	5.43	228	0.0112
S0->S9	5.60	222	0.3246
S0->S10	5.66	219	0.1351

DAP27_edge-out	E_{vert} (eV)	E_{vert} (nm)	*f*
S0->S1	4.04	307	0.3030
S0->S2	4.21	295	0.1182
S0->S3	4.33	286	0.0035
S0->S4	4.70	264	0.0545
S0->S5	4.74	261	0.0005
S0->S6	5.11	243	0.4413
S0->S7	5.37	231	0.0013
S0->S8	5.46	227	0.1291
S0->S9	5.57	222	0.4301
S0->S10	5.77	215	0.0024

Table S13: Possible photoproducts concerning peroxide formation: relevant electronic singlet transitions from S_0, giving vertical energies E_{vert}, oscillator strengths *f* for the compounds under study at the TD-DFT level (D3-B3LYP /6-311G(d,p)//D3-B3LYP/6-311G(d,p) PCM: DCM) [right].

DAP16_top-in	E_{vert} (eV)	E_{vert} (nm)	*f*
S0->S1	3.28	378	0.2135
S0->S2	3.88	320	0.0011
S0->S3	3.94	315	0.1221
S0->S4	4.23	293	0.0012
S0->S5	4.50	276	0.0859
S0->S6	4.70	264	0.0259
S0->S7	4.85	255	0.0002
S0->S8	4.86	255	0.5558
S0->S9	5.10	243	0.0005
S0->S10	5.12	242	0.0619

DAP27_top-out	E_{vert} (eV)	E_{vert} (nm)	*f*
S0->S1	3.48	357	0.2155
S0->S2	3.89	319	0.0033
S0->S3	3.92	316	0.1605
S0->S4	4.27	291	0.0000
S0->S5	4.50	275	0.0003
S0->S6	4.76	260	0.2490
S0->S7	4.86	255	0.0240
S0->S8	5.08	244	0.0027
S0->S9	5.09	244	0.0901
S0->S10	5.25	236	0.7242

DAP16_edge-in	E_{vert} (eV)	E_{vert} (nm)	*f*
S0->S1	2.60	477	0.1229
S0->S2	3.37	368	0.0259
S0->S3	3.51	354	0.0004
S0->S4	3.83	323	0.2709
S0->S5	3.96	313	0.0005
S0->S6	4.18	297	0.0057
S0->S7	4.26	291	0.0592
S0->S8	4.38	283	0.0290
S0->S9	4.51	275	0.0454
S0->S10	4.58	271	0.0002

DAP27_edge-out	E_{vert} (eV)	E_{vert} (nm)	*f*
S0->S1	3.56	348	0.1291
S0->S2	3.72	333	0.2001
S0->S3	3.85	322	0.0019
S0->S4	4.15	299	0.0472
S0->S5	4.26	291	0.0000
S0->S6	4.51	275	0.1638
S0->S7	4.71	263	0.0647
S0->S8	4.79	259	0.0006
S0->S9	4.87	254	0.0893
S0->S10	5.00	248	0.1295

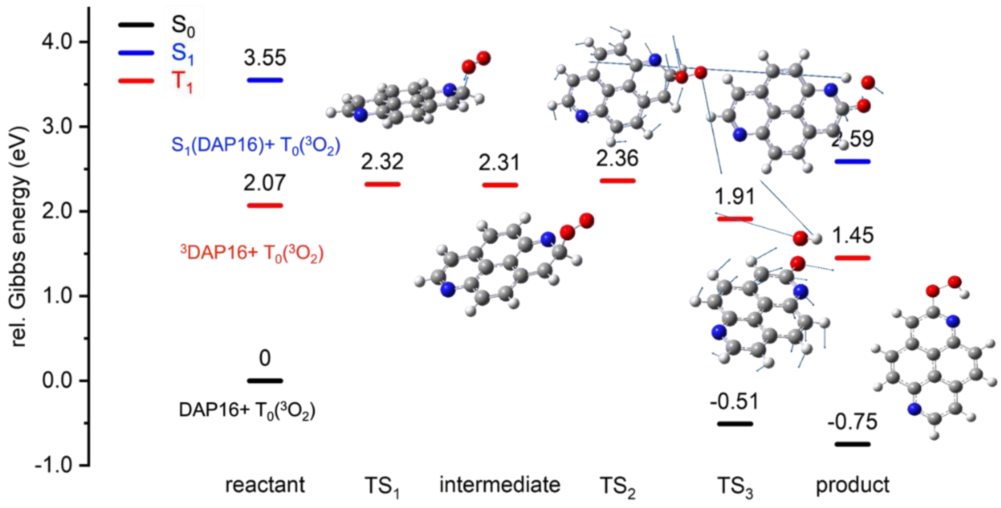

Figure S27: Model reaction pathway showing reactant, transition structures (TS), intermediate structures and products at the TD-DFT level (M06-2X/6-311G(d,p)//M06-2X/6-311G(d,p) PCM: DCM).

References

[1] P. Tavan, K. Schulten, J. Chem. Phys. 1979, 70, 5407.

[2] I. A. Mikhailov, S. Tafur, A. E. Masunov, Phys. Rev. A 2008, 77, 012510.

[3] J. C. Roldao, E. F. Oliviera, B. Milián-Medina, J. Gierschner, D. Roca-Sanjuán, J. Chem. Theor. Comp. 2022, 18, 5449–5458.

[4] J. C. Roldao, E. F. Oliviera, B. Milián-Medina, J. Gierschner, D. Roca-Sanjuán, J. Chem. Phys. 2022, 156, 044102.

[5] E. F. Oliveira, J. Shi, F. C. Lavarda, L. Lüer, B. Milián-Medina, J. Gierschner, J. Chem. Phys. 2017, 147, 034903.

[6] B. Shi, D. Nachtigallová, A. J. A. Aquino, F. B. C. Machado, H. Lischka, J. Chem. Phys. 2019, 150, 124302.

[7] S. Shirai, S. Inagaki, RSC Adv. 2020, 10, 12988.

[8] J. N. Harvey, M. Aschi, H. Schwarz, W. Koch, Theor. Chem. Acc. 1998, 99, 95–99.

[9] T. Lu, sobMECP program, http://sobereva.com/286 (accessed 2023-06-20)
